# Functionalization of *N*-arylglycine esters: electrocatalytic access to C–C bonds mediated by *n*-Bu_4_NI

**DOI:** 10.3762/bjoc.14.35

**Published:** 2018-02-22

**Authors:** Mi-Hai Luo, Yang-Ye Jiang, Kun Xu, Yong-Guo Liu, Bao-Guo Sun, Cheng-Chu Zeng

**Affiliations:** 1 Beijing Key Laboratory of Environmental and Viral Oncology, College of Life Science & Bioengineering, Beijing University of Technology, Beijing 100124, China; 2 Beijing Key Laboratory of Flavor Chemistry, Beijing Technology and Business University, Beijing 100048, China

**Keywords:** C–C formation, electrochemical oxidative functionalization, *n*-Bu_4_NI, redox catalyst

## Abstract

An efficient electrocatalytic functionalization of *N*-arylglycine esters is reported. The protocol proceeds in an undivided cell under constant current conditions employing the simple, cheap and readily available *n*-Bu_4_NI as the mediator. In addition, it is demonstrated that the mediated process is superior to the direct electrochemical functionalization.

## Introduction

**Scheme 1:**
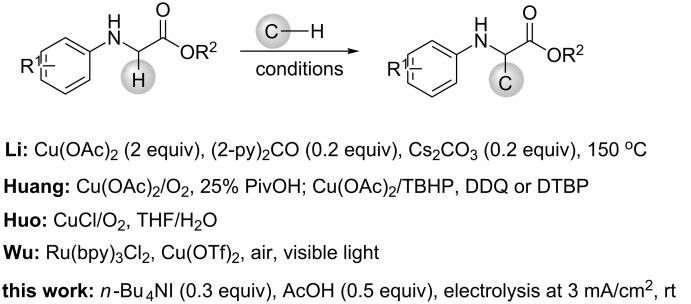
Cross dehydrogenative coupling of *N*-arylglycine esters with C–H nucleophiles.

The oxidative cross dehydrogenative coupling (CDC) of two C–H bonds has emerged as an versatile and powerful strategy for forming new C–C bonds in organic chemistry due to its step and atom economic characteristic as well as avoiding the prefunctionalization of substrates [1–5]. Most of the CDC reactions occur between the benzylic C–H bonds and α-C–H bonds adjacent to a heteroatom (N or O) [6–10]. However, the oxidative C–C formation of secondary amines, especially amino acids has been more important for studying properties and functions of natural and non-natural amino acids [11]. Consequently, efficient and selective construction of C–C bonds of amino acids has always been paid much attention in industrial and academic setting and many advances have been made [12–17]. Li et al. first reported the functionalization of glycine derivatives with malonates using a stoichiometric quantity of Cu(OAc)_2_ as catalyst and oxidant [12]. Later on, arylation, vinylation and alkynylation of glycine derivatives were also accomplished by the same group (Scheme 1) [13]. Using the Cu(OAc)_2_/pyrrolidine dual catalysts system, Huang developed the oxidative cross coupling of glycine derivatives with acetone in the presence of TBHP or DDQ as terminal oxidants [14]. The protocol was also extended to reactions with 2-alkylquinoline [15] and phenols [16] using O_2_ and di-*tert*-butyl peroxide (DTBP) as oxidant, respectively (Scheme 1). A CuCl-catalyzed oxidative cross coupling of glycine derivatives with indoles has been developed by Hou et al., wherein simple copper salts were used as catalysts and oxygen as the co-oxidant (Scheme 1) [17].

Alternatively, photocatalytic versions of CDC reactions of glycine derivatives with C-nucleophiles were also developed [18–19]. For example, combining the visible light catalyst Ru(bpy)_3_Cl_2_, and the transition metal catalyst Cu(OTf)_2_, Wu and co-workers achieved aerobic oxidative coupling of secondary amines with β-keto esters to form C(sp^3^)–C(sp^3^) bonds (Scheme 1) [18].

Although much advance has been made for the functionalization of glycine derivatives, most of these strategies mentioned above require stoichiometric or excess amounts of chemical oxidants and transition metal (photo)catalysts. The utilization of stoichiometric or excess amounts of chemical oxidants results in producing waste, which is not atomic economically and environmentally benign. In addition, over-oxidation of products likely occurs in the presence of excess amounts of oxidant. On the other hand, the toxicity of residual traces of transition metal (photo)catalyst in products is also highly concerned. Consequently, metal-free and environmentally friendly oxidative C–C bond formation is highly desired.

Electrochemistry has proved to be an environmentally benign method to achieve the formation of a new chemical bond and a functional group conversion by using electrons as redox reagent rather than terminal chemicals [20–27]. In this context, we have applied simple halide ions as redox catalysts to achieve electrochemical C–H bond functionalization, leading to the formation of new C–C, C–N, C–O and C–S bonds [28–31]. Herein, we report the electrochemical α-C–H functionalization of *N*-arylglycine esters with C–H nucleophiles using *n*-Bu_4_NI as redox catalyst (Scheme 1). The chemistry was performed in an undivided cell under constant current electrolysis. It was observed that *n*-Bu_4_NI promotes the reaction dramatically and higher yields of α-functionalized products were afforded compared with the direct electrolysis.

## Results and Discussion

Initially, *N*-arylglycine ester **1a** and C–H nucleophile 1,3,5-trimethoxybenzene (**2a**) were chosen as model compounds to optimize the electrolytic conditions. As shown in Table 1, when constant current electrolysis (CCE) of **1a** and **2a** was performed in an undivided cell equipped with 0.1 M LiClO_4_ in CH_3_CN in the presence of AcOH using two graphite plates as anode and cathode, the desired product **3aa** was isolated in 27% yield (Table 1, entry 1). Replacing CH_3_CN by other solvents, such as methanol, ethanol or CH_2_Cl_2_ failed to improve the reaction efficiency and only trace amounts of **3aa** were detected (Table 1, entries 2–4). Further solvent screening disclosed that a 1:2 ratio of CH_3_CN and CH_2_Cl_2_ was better, giving **3aa** in 66% yield (Table 1, entries 5 and 6). Exploring the influence of current density on the CDC reaction indicated that 3 mA/cm^2^ was suitable; lower or higher current density led to a slightly lower yields of **3aa** (entry 5, vs entries 7 and 8). It was observed that the additive plays an important role. Among several additives examined, AcOH was proved to be the best, although TFA also gave comparable yields of **3aa** (Table 1, entries 5 and 11–13), whereas, it gave only 38% yield of **3aa** when the reaction was carried out in the absence of AcOH (Table 1, entry 9). Investigation of the anode proved that graphite was superior to Pt and DSA (Dimensionally Stable Anode, Table 1, entries 14 and 15). Finally, to further improve the reaction efficiency, several halide-containing mediators as redox catalyst were evaluated. To our delight, when *n*-Bu_4_NI was utilized as a redox catalyst, the yield of **3aa** increased to 81% (Table 1, entries 16–19).

**Table 1:** Optimization of reaction conditions^a^.

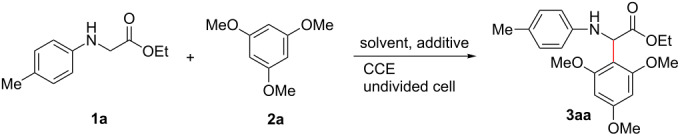						

entry	solvent	mediator (equiv)	*J* (mA/cm^2^)	additive (equiv)	cathode/anode	yield (%)^b^

**1**	CH_3_CN	–^c^	3	AcOH (0.5)	C/C	27
**2**	MeOH	–^c^	3	AcOH (0.5)	C/C	trace
**3**	EtOH	–^c^	3	AcOH (0.5)	C/C	trace
**4**^d^	CH_2_Cl_2_	–^c^	3	AcOH(0.5)	C/C	trace
**5**	CH_3_CN/CH_2_Cl_2_ (1:2)	–^c^	3	AcOH (0.5)	C/C	66
**6**	CH_3_CN/CH_2_Cl_2_ (2:1)	–^c^	3	AcOH (0.5)	C/C	56
**7**	CH_3_CN/CH_2_Cl_2_ (1:2)	–^c^	1	AcOH (0.5)	C/C	47
**8**	CH_3_CN/CH_2_Cl_2_(1:2)	–^c^	5	AcOH (0.5)	C/C	50
**9**	CH_3_CN/CH_2_Cl_2_ (1:2)	–^c^	3	AcOH (0.0)	C/C	38
**10**	CH_3_CN/CH_2_Cl_2_ (1:2)	–^c^	3	AcOH (1.0)	C/C	56
**11**	CH_3_CN/CH_2_Cl_2_ (1:2)	–^c^	3	TFA (0.5)	C/C	60
**12**	CH_3_CN/CH_2_Cl_2_ (1:2)	–^c^	3	H_2_SO_4_ (0.5)	C/C	13
**13**	CH_3_CN/CH_2_Cl_2_ (1:2)	–^c^	3	Na_2_CO_3_ (0.5)	C/C	40
**14**	CH_3_CN/CH_2_Cl_2_ (1:2)	–^c^	3	AcOH (0.5)	C/Pt	48
**15**	CH_3_CN/CH_2_Cl_2_ (1:2)	–^c^	3	AcOH (0.5)	C/DSA	41
**16**	CH_3_CN/CH_2_Cl_2_ (1:2)	*n*-Bu_4_NI (0.3)	3	AcOH (0.5)	C/C	81
**17**	CH_3_CN/CH_2_Cl_2_ (1:2)	*n*-Bu_4_NBr (0.3)	3	AcOH (0.5)	C/C	48
**18**	CH_3_CN/CH_2_Cl_2_ (1:2)	NH_4_I (0.3)	3	AcOH (0.5)	C/C	54
**19**	CH_3_CN/CH_2_Cl_2_ (1:2)	NH_4_Br (0.3)	3	AcOH (0.5)	C/C	20

^a^Conditions: **1a** (1.0 mmol), **2a** (1.2 mmol) in 15 mL solution, LiClO_4_ (0.1 M), room temperature, electrode C represents graphite plate. ^b^Isolated yield. ^c^Without mediator. ^d^*n*-Bu_4_NBF_4_ was used as supporting electrolyte.

**Scheme 2:**
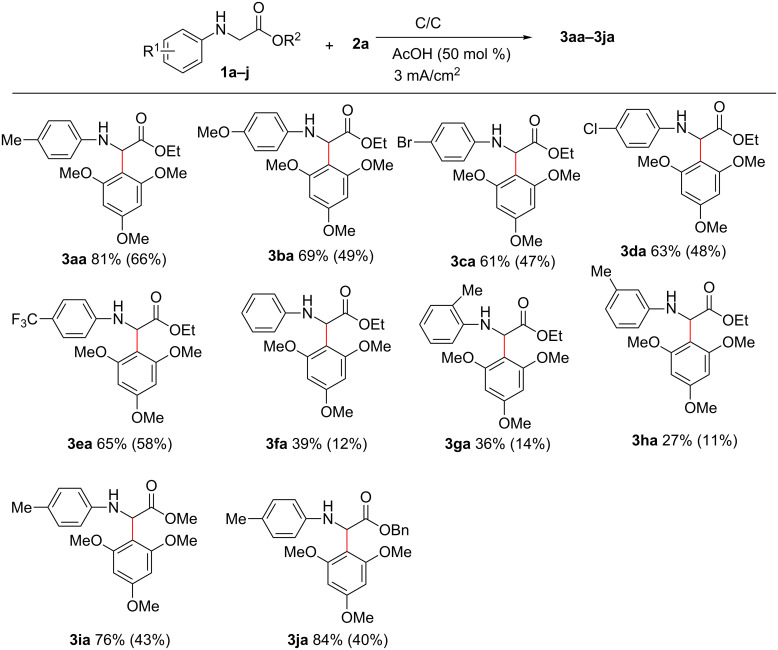
Electrochemical CDC reaction of **2a** and various *N*-arylglycine esters. Reaction conditions for the indirect electrolysis: **1** (0.5 mmol), **2a** (0.6 mmol), *n*-Bu_4_NI (30 mol %), 0.1 M LiClO_4_/CH_3_CN (5 mL) and CH_2_Cl_2_ (10 mL), AcOH (50 mol %), current density (3 mA/cm^2^), graphite anode and cathode, at room temperature; reaction conditions for direct electrolysis: **1** (0.5 mmol), **2a** (0.6 mmol), 0.1 M LiClO_4_/CH_3_CN (5 mL) and CH_2_Cl_2_ (10 mL), AcOH (50 mol %), current density (3 mA/cm^2^), graphite anode and cathode, at room temperature; yields in parenthesis obtained from direct electrolysis.

On the basis of the screening of reaction conditions, we could conclude that the electrocatalytic oxidative coupling should be performed in a mixed solution of CH_3_CN and CH_2_Cl_2_ (v/v = 1:2), in the presence of *n*-Bu_4_NI (30 mol %) as the mediator, AcOH (50 mol %) as the additive and using graphite plate as electrodes under constant current at 3 mA/cm^2^. With the optimized conditions in hand, we then investigated the scope of *N*-arylglycine esters in the reaction with **2a**. As a comparison, direct electrochemical coupling of *N*-arylglycine esters with **2a** was also performed. As shown in Scheme 2, substituents including either electron-donating groups (such as methyl and methoxy) or electron-withdrawing groups (Cl, Br and CF_3_) in the 4-position of the aryl group were tolerated and gave moderate to good yields of **3ba**–**3ea** (61–69% yields), whereas, only 47–58% yields of the desired **3ba**–**3ea** were isolated under the direct electrolytic conditions. However, **3fa** was afforded in 39% and 12% yields, respectively, when the electrochemical reaction was performed in the presence or absence of *n*-Bu_4_NI as the redox catalyst. The reason for the low yield of **3fa** is not clear yet. Steric factors seem to play an important role in the electrochemical CDC reaction of *N*-arylglycine esters with **2a**. When the methyl group was situated at 2- or 3-position of the aniline, instead of in the 4-position, the corresponding products **3ga** and **3ha** were afforded in 36% and 27% yields, respectively. However, **3ga** and **3ha** were isolated in 14% and 11% yields, respectively, under the direct electrolytic conditions. In the cases of *N*-arylglycine methyl ester **1i** and *N*-arylglycine benzyl ester **1j**, the electrocatalytic functionalization afforded excellent yields of **3ia** (76%) and **3ja** (84%), whereas, less efficiency (40–43% yields) was observed without the assistance of *n*-Bu_4_NI.

**Scheme 3:**
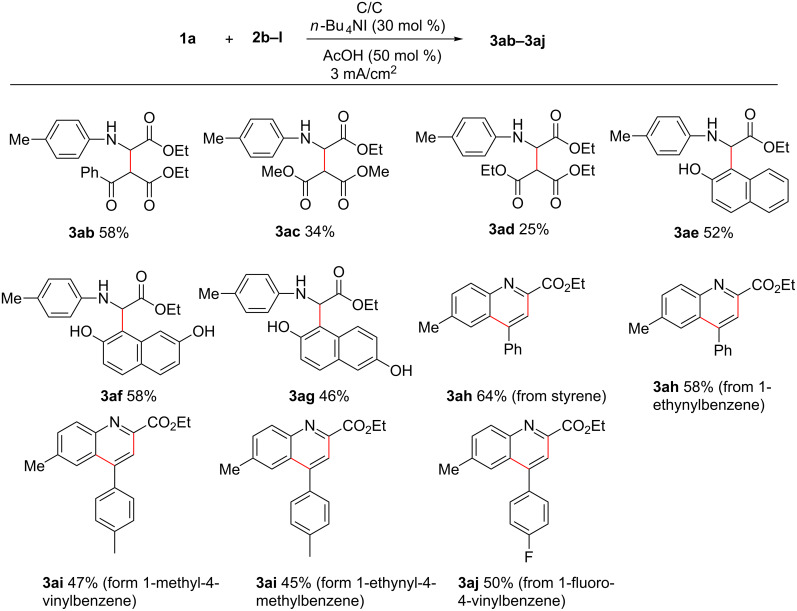
Scope of **2** using *n*-Bu_4_NI as mediator. Reaction conditions:**1a** (0.5 mmol), **2** (0.6 mmol), *n*-Bu_4_NI (30 mol %), 0.1 M LiClO_4_/CH_3_CN (5 mL) and CH_2_Cl_2_ (10 mL), AcOH (50 mol %), current density (3 mA/cm^2^), graphite anode and cathode. Isolated yields are given.

Next, the reactivity of different C–H nucleophiles was also investigated. As shown in Scheme 3, β-keto ester and malonates worked well to afford the desired products **3ab**–**3ad** in moderate yields. In addition, naphthanols were also compatible in this transformation, giving the corresponding products **3ae**–**3ag** in good yields. Notably, when styrene and 1-ethynylbenzene were subjected to reaction with **1a** under the standard indirect electrolytic conditions, quinoline-2-carboxylate **3ah** was isolated in 64% and 58% yield, respectively. Substituted styrenes and 1-ethynylbenzene were also tolerated well, giving corresponding products **3ai**–**3aj** in 45–50% yields. The formations of **3ah**–**3aj** likely derives from an azo-Diels–Alder reaction of styrene or ethynylbenzene with an imine intermediate, in situ generated from anodic oxidation of **1a**, followed by additional oxidation [32–36].

**Scheme 4:**
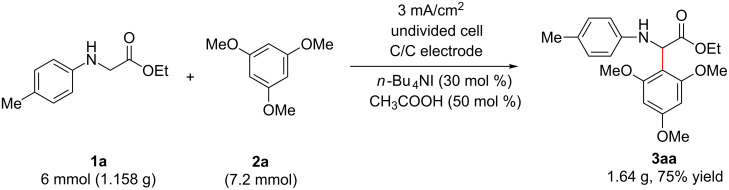
Scaling up.

To prove the practicability of the protocol, a scaled-up reaction was also carried out. As illustrated in Scheme 4, when 6 mmol of ethyl *p*-tolylglycinate (**1a**) was allowed to react with 1,3,5-trimethoxybenzene (**2a**) under the standard conditions, adduct **3aa** was isolated in a 75% yield, without obvious losing of yield.

**Scheme 5:**
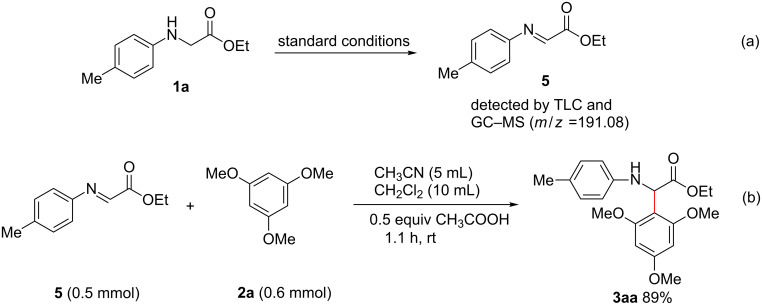
Control experiments.

To better understand the reaction mechanism, control experiments were performed. As shown in Scheme 5, the anodic oxidation of **1a** in the absence of a C–H nucleophile under the standard conditions gives imine intermediate product **5**, which was detected by TLC and GC–MS. Moreover, when separated synthesized **5** was subjected to react with **2a**, the corresponding product **3aa** was isolated in 89% yield. These control experiments indicate that **5** is a possible reaction intermediate.

**Scheme 6:**
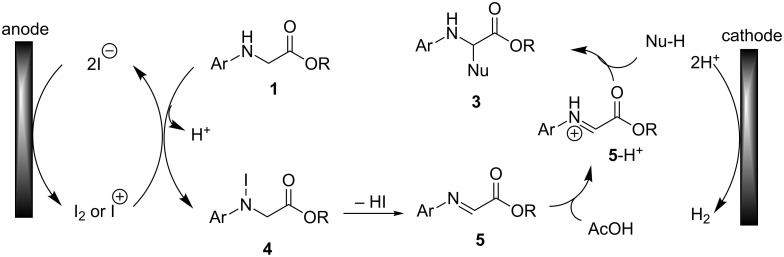
A plausible mechanism for the electrocatalytic cross dehydrogenative coupling of *N*-arylglycine esters with C-nucleophiles.

Based on these control experiments described above, as well as related references [4], a plausible mechanism for the electrocatalytic cross dehydrogenative coupling of *N*-arylglycine esters **1** with C–H nucleophiles **2** is outlined in Scheme 6. The anodic oxidation of iodide generates the active species I_2_ or I^+^. Followed by a homogeneous reaction with *N*-arylglycine esters, *N*-iodo intermediate **4** was generated. Eliminating a molecule of HI affords imine intermediate **5**. In the presence of acetic acid, the protonated **5**-H^+^ undergoes nucleophilic addition with C–H nucleophiles **2** to give the desired products **3**.

## Conclusion

In summary, an efficient electrocatalytic cross dehydrogenative coupling of arylglycine esters with C–H nucleophiles has been developed. This protocol employs simple *n*-Bu_4_NI as the redox catalyst, avoiding utilization of transition metals and excess amounts of external oxidant, thereby providing an environmentally benign method to the CDC reaction. In addition, it was observed that the electrocatalytic process is superior to the direct electrolysis. Further application of this electrochemical protocol for the formation of new C–C bonds is still on the way in our group.

## Experimental

### Instruments and reagents

All melting points were measured with an electrothermal melting point apparatus and are uncorrected. NMR spectra were recorded using a 400 MHz or 300 MHz spectrometer (400 MHz ^1^H frequency, 100 MHz ^13^C frequency; 300 MHz ^1^H frequency, 75 MHz ^13^C frequency). Chemical shifts are given as δ values (internal standard: TMS). Coupling constants are reported in Hz. All starting materials and solvents were obtained from commercial sources and used without further purification. Products were purified by chromatography on silica gel (petroleum ether/EtOAc).

#### Typical procedure for the electrocatalytic cross dehydrogenative coupling of *N*-arylglycine ester and C–H nucleophiles

An undivided cell was equipped with a carbon anode (2 × 2 cm^2^) and a carbon cathode (2 × 2 cm^2^) and connected to a DC regulated power supply. To the cell was added *N*-arylglycine ester (0.5 mmol), C–H nucleophiles (0.6 mmol), *n*-Bu_4_NI (0.15 mmol) and 5 mL of 0.1 M LiClO_4_/CH_3_CN and 10 mL CH_2_Cl_2_. The mixture was electrolyzed using constant current conditions (≈3 mA/cm^2^) at room temperature under magnetic stirring. When TLC analysis indicated that the electrolysis was complete (witnessed by the disappearance of the *N*-arylglycine ester), the solvent was removed under reduced pressure. The residue was poured into a saturated aqueous solution of Na_2_S_2_O_3_ and the product was then extracted with DCM (3 × 20 mL), dried over Na_2_SO_4_, and concentrated in vacuo. The residue was purified by column chromatography on silica gel using a mixture of petroleum ether/EtOAc (v/v = 3:1) as eluent to afford the desired pure product.

#### Typical procedure for the direct electrochemical cross dehydrogenative coupling of *N*-arylglycine ester and C–H nucleophiles

The procedure was identical to that of electrocatalytic synthesis, but without the addition of *n*-Bu_4_NI as the mediator.

## Supporting Information

File 1 General procedure and analytical data.
