# Supplementary material for: Functionalization of N-arylglycine esters: electrocatalytic access to C–C bonds mediated by n-Bu4NI
Source: Beilstein J Org Chem. 2018 Feb 22;14:499–505. doi: 10.3762/bjoc.14.35 (PMC5827798; doi:10.3762/bjoc.14.35)
Supplement: File 1 — General procedure and analytical data. [file Beilstein_J_Org_Chem-14-499-s001.pdf]

Supporting Information

for

Functionalization of *N*-arylglycine esters: electrocatalytic access to C–C bonds mediated by *n*-Bu_4NI

Mi-Hai Luo,[1] Yang-Ye Jiang,[1] Kun Xu,[1] Yong-Guo Liu,[2] Bao-Guo Sun[2] and Cheng-Chu Zeng[*,1]

Address: [1]Beijing Key Laboratory of Environmental and Viral Oncology, College of Life Science & Bioengineering, Beijing University of Technology, Beijing 100124, China and [2]Beijing Key Laboratory of Flavor Chemistry, Beijing Technology and Business University, Beijing 100048, China

Email: Cheng-Chu Zeng - zengcc@bjut.edu.cn

*Corresponding author

Content

Content………………………………………………………………………. …………………. …………...…S2

General procedure for the synthesis of *N*-ethyl acetate-substituted anilines.[1] S4

Compounds characterization S4

Spectra of prepared compounds S9

^{1}H NMR spectra of ethyl 2-(4-tolylamino)-2-(2,4,6-trimethoxyphenyl)acetate (**3aa**) S9

^{1}H NMR spectra of ethyl 2-((4-methoxyphenyl) amino)-2-(2,4,6-trimethoxyphenyl)acetate (**3ba**) S10

HRMS spectra of ethyl 2-((4-methoxyphenyl) amino)-2-(2,4,6-trimethoxyphenyl)acetate (**3ba**) S11

^{1}H NMR spectra of ethyl2-(4-bromophenylamino)-2-(2,4,6-trimethoxyphenyl)acetate (**3ca**) S12

^{13}C NMR spectra of ethyl2-(4-bromophenylamino)-2-(2,4,6-trimethoxyphenyl)acetate (**3ca**) S12

^{1}H NMR spectra of ethyl 2-(4-chlorophenylamino)-2-(2,4,6-trimethoxyphenyl)acetate (**3da**) S13

^{13}C NMR spectra of ethyl 2-(4-chlorophenylamino)-2-(2,4,6-trimethoxyphenyl)acetate (**3da**) S13

^{1}H NMR spectra of ethyl 2-((4-(trifluoromethyl)phenyl)amino)-2-(2,4,6-trimethoxyphenyl)acetate (**3ea**) . S14

^{13}C NMR spectra of ethyl2-((4-(trifluoromethyl)phenyl)amino)-2-(2,4,6-trimethoxyphenyl)acetate (**3ea**) . S14

HRMS spectra of ethyl 2-((4-(trifluoromethyl)phenyl)amino)-2-(2,4,6-trimethoxyphenyl)acetate (**3ea**) S15

^{1}H NMR spectra of ethyl 2-(phenylamino)-2-(2,4,6-trimethoxyphenyl)acetate (**3fa**) S16

^{13}C NMR spectra of ethyl 2-(phenylamino)-2-(2,4,6-trimethoxyphenyl)acetate (**3fa**) S16

^{1}H NMR spectra of ethyl 2-(2-tolylamino)-2-(2,4,6-trimethoxyphenyl)acetate (**3ga**) S17

^{13}C NMR spectra of ethyl 2-(2-tolylamino)-2-(2,4,6-trimethoxyphenyl)acetate (**3ga**) S17

^{1}H NMR spectra of ethyl 2-(3-tolylamino)-2-(2,4,6-trimethoxyphenyl)acetate (**3ha**) S18

^{13}C NMR spectra of ethyl 2-(3-tolylamino)-2-(2,4,6-trimethoxyphenyl)acetate (**3ha**) S18

^{1}H NMR spectra of methyl 2-(4-tolylamino)-2-(2,4,6-trimethoxyphenyl)acetate (**3ia**) S19

^{13}C NMR spectra of methyl 2-(4-tolylamino)-2-(2,4,6-trimethoxyphenyl)acetate (**3ia**) S19

^{1}H NMR spectra of benzyl 2-(*p*-tolylamino)-2-(2,4,6-trimethoxyphenyl)acetate (**3ja**) S20

^{13}C NMR spectra of benzyl 2-(*p*-tolylamino)-2-(2,4,6-trimethoxyphenyl)acetate (**3ja**) S20

HRMS spectra of benzyl 2-(*p*-tolylamino)-2-(2,4,6-trimethoxyphenyl)acetate (**3ja**) S21

^{1}H NMR spectra of diethyl 2-benzoyl-3-(*p*-tolylamino)succinate (**3ab**) S22

^{13}C NMR spectra of diethyl 2-benzoyl-3-(*p*-tolylamino)succinate (**3ab**) S22

^{1}H NMR spectra of 2-ethyl 1,1-dimethyl 2-(*p*-tolylamino)ethane-1,1,2-tricarboxylate (**3ac**) S23

^{13}C NMR spectra of 2-ethyl 1,1-dimethyl 2-(*p*-tolylamino)ethane-1,1,2-tricarboxylate (**3ac**) S23

^{1}H NMR spectra of triethyl 2-(p-tolylamino)ethane-1,1,2-tricarboxylate (**3ad**) S24

^{13}C NMR spectra of triethyl 2-(*p*-tolylamino)ethane-1,1,2-tricarboxylate (**3ad**) S24

^{1}H NMR spectra of ethyl 2-(4-tolylamino)-2-(2-hydroxynaphthalen-1-yl)acetate (**3ae**) S25

^{13}C NMR spectra of ethyl 2-(4-tolylamino)-2-(2-hydroxynaphthalen-1-yl)acetate (**3ae**) S25

^{13}C NMR spectra of ethyl 2-(2,7-dihydroxynaphthalen-1-yl)-2-(*p*-tolylamino)acetate (**3af**) S26

HRMS spectra of ethyl 2-(2,7-dihydroxynaphthalen-1-yl)-2-(*p*-tolylamino)acetate (**3af**)........................... S27

^{1}H NMR spectra of ethyl 2-(2,6-dihydroxynaphthalen-1-yl)-2-(*p*-tolylamino)acetate (**3ag**) S28

^{13}C NMR spectra of ethyl 2-(2,6-dihydroxynaphthalen-1-yl)-2-(*p*-tolylamino)acetate (**3ag**)...................... S28

HRMS spectra of ethyl 2-(2,6-dihydroxynaphthalen-1-yl)-2-(*p*-tolylamino)acetate (**3ag**) S29

^{1}H NMR spectra of ethyl 6-methyl-4-phenylquinoline-2-carboxylate (**3ah**) S30

^{13}C NMR spectra of ethyl 6-methyl-4-phenylquinoline-2-carboxylate (**3ah**) S30

^{1}H NMR spectra of ethyl 6-methyl-4-(p-tolyl)quinoline-2-carboxylate (**3ai**) S31

^{13}C NMR spectra of ethyl 6-methyl-4-(p-tolyl)quinoline-2-carboxylate (**3ai**) S31

^{1}H NMR spectra of ethyl 4-(4-fluorophenyl)-6-methylquinoline-2-carboxylate (**3aj**) S32
^{13}C NMR spectra of ethyl 4-(4-fluorophenyl)-6-methylquinoline-2-carboxylate (**3aj**) S32
References..S33

General procedure for the synthesis of *N*-ethyl acetate-substituted anilines.[1]

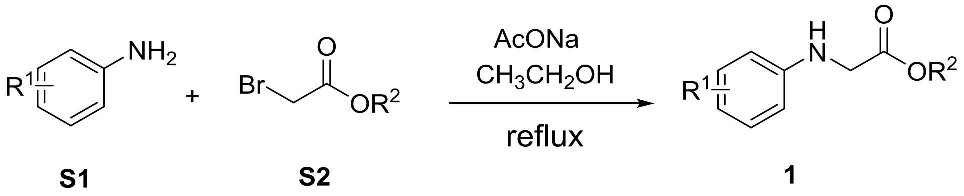

In an ordinary vial equipped with a magnetic stirring bar, aniline **S1** (10 mmol,1.0 equiv), ethyl bromoacetate **S2** (10 mmol,1.0 equiv), sodium acetate (10 mmol,1.0 equiv), and ethanol (6.6 M) were added. After the reaction mixture was stirred at 85−90 °C overnight, the solvent was removed in vacuo. The residue was dissolved in DCM and washed with saturated NaCl (aqueous), the aqueous layer was washed two times with DCM, and the combined organic layers were dried over $MgSO_4$. Finally, the residue was purified by column chromatography.

Compounds characterization

Ethyl 2-(4-tolylamino)-2-(2,4,6-trimethoxyphenyl)acetate (**3aa**) [2] White solid; mp: 99-101 ^{o}C; ^{1}HNMR(400 MHz, $CDCl_3$), δ (ppm): 1.19 (t, J = 7.2 Hz, 3H), 2.22 (s, 3H), 3.81 (s, 3H), 3.85 (s, 6H), 4.11- 4.25 (m, 2H), 4.79 (br, 1H), 5.67 (s, 1H), 6.13 (s, 2H), 6.68 (d, J = 8.4 Hz, 2H), 6.96 (d, J = 8.0 Hz, 2H); ^{13}C NMR(100 MHz, $CDCl_3$), δ (ppm): 14.2, 20.4, 51.4, 55.3, 55.9, 61.0, 91.0, 108.9, 114.1, 126.8, 129.5, 145.2, 158.8, 160.9, 173.1.

Ethyl 2-((4-methoxyphenyl)amino)-2-(2,4,6-trimethoxyphenyl)acetate (**3ba**) White solid; mp: 76-77 ^{o}C; ^{1}H NMR (400 MHz, $CDCl_3$), δ (ppm): 1.16 (t, J = 7.2 Hz, 3H), 3.71 (s, 3H), 3.78 (s, 3H), 3.82 (s, 6H), 4.08-4.26 (m, 2H), 5.58 (s, 1H), 6.11 (s, 2H), 6.64 (s, 4H); ^{13}C NMR (100 MHz, $CDCl_3$), δ (ppm): 14.2, 52.3, 55.3, 55.7, 55.9, 61.0, 91.0, 108.8, 114.6, 115.6, 141.7, 152.4, 158.8, 160.9, 173.2;HRMS (ESI) m/z calculated for $C_{20}H_{25}NO_6$ $(M+Na)^+$ 398.1574, Found 398.1578.

Ethyl 2-(4-bromophenylamino)-2-(2,4,6-trimethoxyphenyl)acetate (**3ca**) [2] White solid; mp:

113-115 ^{o}C; ^{1}HNMR(300 MHz, CDCl3),δ (ppm): 1.18 (t, *J* = 7.2 Hz, 3H), 3.80 (s, 3H), 3.84 (s, 6H), 4.11-4.24 (m, 2H), 4.93 (br, 1H), 5.61 (s, 1H), 6.12 (s, 2H), 6.61 (d, *J* = 9.0 Hz, 2H), 7.20 (d, *J* = 8.7 Hz, 2H); ^{13}C NMR(100 MHz, $CDCl_3$), *δ* (ppm): 14.2, 50.9, 55.3, 55.9, 61.2, 91.0, 108.0, 109.2, 115.4, 131.7, 146.4, 158.7, 161.1, 172.6.

Ethyl 2-(4-chlorophenylamino)-2-(2,4,6-trimethoxyphenyl)acetate (**3da)** [2] White solid; mp: 106-107 ^{o}C; ^{1}H NMR (400 MHz, $CDCl_3$), *δ* (ppm): 1.17 (t, *J* = 7.2 Hz, 3H), 3.79 (s, 3H), 3.83 (s, 6H), 4.09-4.23 (m, 2H), 5.60 (s, 1H), 6.11 (s, 2H), 6.64 (d, *J* = 8.8 Hz, 2H), 7.05 (d, *J* = 8.8 Hz, 2H); ^{13}C NMR (100 MHz, $CDCl_3$), *δ* (ppm): 14.2, 51.0, 55.3, 55.9, 61.2, 91.0, 108.1, 114.9, 122.1, 128.8, 146.0, 158.7, 161.1, 172.7;

Ethyl 2-((4-(trifluoromethyl)phenyl)amino)-2-(2,4,6-trimethoxyphenyl)acetate (**3ea)** White solid; mp: 99-100°C; ^{1}H NMR (400 MHz, $CDCl_3$), *δ* (ppm): 1.20 (t, *J* = 7.2 Hz, 3H), 3.82 (s, 3H), 3.87 (s, 6H), 4.12-4.27 (m, 2H), 5.23 (d, *J* = 7.6 Hz, 1H), 5.70 (d, *J* = 7.6 Hz, 1H), 6.15 (s, 2H), 6.73 (d, *J* = 7.6 Hz, 2H), 7.36 (d, *J* = 8.4 Hz, 2H); ^{13}C NMR (100 MHz, $CDCl_3$), *δ* (ppm): 14.2, 50.3, 55.3, 55.9, 61.3, 91.0, 107.8, 112.5, 118.8 (q, J_{CF} = 32.5 Hz), 125.0 (q, J_{CF}= 268.7 Hz), 126.4 (q, J_{CF} = 3.8 Hz), 149.7, 158.7, 161.2, 172.3; HRMS (ESI) m/z calculated for $C_{20}H_{22}F_3NO_5$ $(M+Na)^+$436.1342, Found 436.1338.

Ethyl 2-(phenylamino)-2-(2,4,6-trimethoxyphenyl)acetate (**3fa**) [2] White solid; mp: 106-108 ^{o}C; ^{1}HNMR(300 MHz, CDCl3), δ (ppm):1.18 (t, *J* = 7.2 Hz, 3H), 3.80 (s, 3H), 3.85 (s, 6H), 4.11-4.24 (m, 2H), 5.69 (s, 1H), 6.13 (s, 2H), 6.67 (t, *J* = 7.2 Hz, 1H), 6.74 (d, *J* = 7.8 Hz, 2H), 7.14(t, *J* = 7.5 Hz,2H);^{13}C NMR(100 MHz, $CDCl_3$), *δ* (ppm): 14.2, 51.0, 55.3, 55.9, 61.1, 91.0, 108.7, 113.8, 117.6, 129.0, 147.4, 158.8, 160.9, 173.0.

Ethyl 2-(2-tolylamino)-2-(2,4,6-trimethoxyphenyl)acetate (**3ga**) [2] White solid; mp: 104-106 ^{o}C;^{1}HNMR(400 MHz, CDCl3), δ (ppm): 1.17 (t, *J* = 7.2 Hz, 3H), 2.20 (s, 3H), 3.79 (s, 3H), 3.85 (s, 6H), 4.12-4.22 (m, 2H), 5.74 (s, 1H), 6.12 (s, 2H), 6.58-6.61 (m, 1H), 6.77 (d, *J* = 8.0 Hz, 1H), 6.99-7.05 (m, 2H); ^{13}C NMR (100 MHz, CDCl3), δ (ppm): 14.2, 17.5, 51.0, 55.3, 55.9, 61.1, 91.0, 108.7, 111.0, 117.1, 122.6, 126.8, 130.0, 145.4, 158.8, 160.9, 173.0.

Ethyl 2-(3-tolylamino)-2-(2,4,6-trimethoxyphenyl)acetate (**3ha**) [2] White solid; mp: 100-102 ^{o}C; ^{1}H NMR (400 MHz, $CDCl_3$), δ (ppm): 1.19 (t, *J* = 7.2 Hz, 3H), 2.26 (s, 3H), 3.81 (s, 3H), 3.86 (s, 6H), 4.12-4.24 (m, 2H), 4.93 (br, 1H), 5.68 (s, 1H), 6.13 (s, 2H), 6.50 (d, *J* = 7.2 Hz, 1H), 6.55-6.58 (m, 2H), 7.02 (t, *J* = 8.0 Hz, 1H); ^{13}C NMR (100 MHz, $CDCl_3$), δ (ppm): 14.2,

21.6, 50.9, 55.3, 55.8, 61.0, 91.0, 108.8, 110.8, 114.5, 118.5, 128.8, 138.6, 147.3, 158.7, 160.9, 173.0.

Methyl 2-(4-tolylamino)-2-(2,4,6-trimethoxyphenyl)acetate (**3ia**) [2] White solid; mp: 162-163 ^{o}C; ^{1}HNMR(300 MHz, $CDCl_3$), δ (ppm): 2.21 (s, 3H), 3.69 (s, 3H), 3.80 (s, 3H), 3.85 (s, 6H), 5.69 (s, 1H), 6.13 (s, 2H), 6.67 (d, *J* = 8.4 Hz, 2H), 6.95 (d, *J* = 8.1 Hz, 2H); ^{13}C NMR (100 MHz, CDCl3), *δ* (ppm): 20.4, 51.2, 52.3, 55.3, 55.9, 91.0, 108.5, 114.1, 126.9, 129.6, 145.0, 158.8, 161.0, 173.6;

Benzyl 2-(p-tolylamino)-2-(2,4,6-trimethoxyphenyl)acetate (**3ja**) White solid; mp: 128-129 ^{o}C;^{1}HNMR(300 MHz, $CDCl_3$), δ (ppm): 2.22 (s, 3H), 3.72 (s, 6H), 3.80 (s, 3H), 4.80 (br, 1H), 5.07 (d, *J* = 12.6 Hz, 1H), 5.25 (d, *J* = 12.3 Hz, 1H), 5.76 (s, 1H), 6.10 (s, 2H), 6.68 (d, *J* = 8.1 Hz, 2H), 6.96 (d, *J* = 8.1 Hz, 2H), 7.19-7.21 (m, 2H), 7.28-2.29 (m, 3H); ^{13}CNMR (75 MHz, $CDCl_3$), δ (ppm):20.4, 51.4, 55.3, 55.7, 66.3, 90.8, 108.4, 114.1, 126.9, 127.8, 127.9, 128.3, 129.6, 136.4, 145.1, 158.7, 161.0, 173.0;HRMS (ESI) m/z calculated for $C_{25}H_{27}NO_5$ $(M+Na)^+$444.1781, Found 444.1777.

Diethyl 2-benzoyl-3-(*p*-tolylamino)succinate (**3ab**) [3] Yellow liquid; ^{1}H NMR (400 MHz, $CDCl_3$), *δ* (ppm): 1.12-1.23 (m, 6H), 2.25 (s, 3H), 4.12-4.20 (m, 4H), 4.33-4.60 (br, 1H), 4.87-5.07 (m, 2H), 6.63 (d, *J* = 8.0 Hz, 1H), 6.69 (d, *J* = 8.0 Hz, 1H), 6.98-7.01 (m, 2H), 7.46-7.52 (m, 2H), 7.58-7.61 (m, 1H), 7.95 (d, *J* = 7.6 Hz, 2H); ^{13}CNMR (100 MHz, $CDCl_3$), δ (ppm): 13.87, 13.90, 14.0, 20.41, 20.44, 55.8, 56.8, 57.3, 58.0, 61.63, 61.69, 61.87, 61.92, 114.3, 114.8, 115.3, 128.28, 128.34, 128.42, 128.55, 128.75, 129.7, 129.8, 133.6, 133.7, 136.1, 136.7, 143.9, 144.7, 167.8, 167.9, 171.6, 171.8, 193.0, 194.5.

2-Ethyl 1,1-dimethyl 2-(p-tolylamino)ethane-1,1,2-tricarboxylate (**3ac**) [4] Yellow liquid; ^{1}HNMR (400 MHz, $CDCl_3$), *δ* (ppm): 1.25 (t, *J* = 7.2 Hz, 3H), 2.26 (s, 3H), 3.74 (s, 3H), 3.81 (s, 3H), 4.10 (d, *J* = 5.6 Hz, 1H), 4.15-4.27 (m, 2H), 4.53 (d, *J* = 8.0 Hz,1H), 4.72 (t, *J* = 7.2 Hz, 1H), 6.65 (d, *J* = 8.0 Hz, 2H), 7.02 (d, *J* = 8.0 Hz, 2H); ^{13}CNMR (100 MHz, $CDCl_3$), δ (ppm): 14.0, 20.4, 52.8, 53.9, 57.1, 61.9, 114.2, 128.4, 129.8, 143.8, 167.6, 168.0, 171.0.

Triethyl 2-(*p*-tolylamino)ethane-1,1,2-tricarboxylate (**3ad**) [4] Yellow liquid; ^{1}HNMR (400 MHz, $CDCl_3$), *δ* (ppm): 1.22-1.33 (m, 9H), 2.25 (s, 3H), 4.07 (d, *J* = 5.2 Hz, 1H), 4.18-4.27 (m, 6H), 4.55 (d, *J* = 9.2 Hz, 1H), 4.70-4.73 (m, 1H), 6.66 (d, *J* = 8.4 Hz, 2H), 7.01 (d, *J* = 8.4 Hz, 2H); ^{13}C NMR (100 MHz, $CDCl_3$), δ (ppm): 14.0, 20.4, 54.3, 57.0, 61.75, 61.8, 61.9, 114.2,

128.3, 129.8, 144.0, 167.1, 167.6, 171.1.

Ethyl 2-(*p*-tolylamino)-2-(2-hydroxynaphthalen-1-yl)acetate (**3ae**) [2] Brown oil; ^{1}H NMR (400 MHz, $CDCl_3$), δ (ppm): 1.12 (t, *J* = 7.2 Hz, 3H), 2.22 (s, 3H), 4.07-4.15 (m, 1H), 4.24-4.32 (m, 1H), 5.10 (br, 1H), 5.86 (s, 1H), 6.65 (d, *J* = 8.4 Hz, 2H), 6.94 (d, *J* = 8.4 Hz, 2H), 7.09 (d, *J* = 8.8 Hz, 1H), 7.41 (t, *J* = 7.4 Hz, 1H), 7.58 (t, *J* = 8.0 Hz, 1H), 7.76 (d, *J* = 8.8 Hz, 1H), 7.84 (d, *J* = 8.0 Hz, 1H), 8.14 (d, *J* = 8.8 Hz, 1H), 10.32 (br, 1H);^{13}C NMR (100 MHz, $CDCl_3$), δ (ppm):13.9, 20.5, 57.5, 62.5, 111.4, 116.2, 119.8, 121.9, 123.0, 126.9, 129.0, 129.2, 129.9, 130.6, 130.8, 132.4, 142.9, 156.0, 171.3;

Ethyl 2-(2,7-dihydroxynaphthalen-1-yl)-2-(*p*-tolylamino)acetate (**3af**) Brown oil; ^{1}H NMR (400 MHz, $CDCl_3$), δ (ppm): 1.13 (t, *J* = 6.8 Hz, 3H), 2.21 (s, 3H), 4.10-4.15 (m, 1H), 4.24-4.28 (m, 1H), 5.04 (br, 1H), 5.28 (br, 1H), 5.65 (s, 1H), 6.63 (d, *J* = 8.0 Hz, 2H), 6.89-7.00 (m, 4H), 7.44 (d, *J* = 1.6 Hz, 1H), 7.66 (d, *J* = 9.2 Hz, 1H), 7.71 (t, *J* = 8.8 Hz, 1H), 10.26 (br, 1H); ^{13}C NMR (100 MHz, CDCl3), δ (ppm): 13.9, 20.5, 57.6, 62.5, 104.9, 110.3, 114.6, 116.2, 117.2, 124.5, 129.9, 130.5, 130.8, 130.9, 134.0, 142.8, 154.8, 156.4, 171.4; HRMS (ESI) m/z calculated for $C_{21}H_{21}NO_4(M+Na)^+$374.1363, Found 374.1367.

Ethyl 2-(2,6-dihydroxynaphthalen-1-yl)-2-(*p*-tolylamino)acetate (**3ag**) Brown oil; ^{1}H NMR (400 MHz, $CDCl_3$), δ (ppm): 1.36 (t, *J* = 7.2 Hz, 3H), 2.24 (s, 3H), 4.09 (d, *J* = 18.3 Hz, 1H), 4.30-4.43 (m, 2H), 4.74 (d, *J* = 18.3 Hz, 1H), 5.27 (br, 1H), 6.34 (d, *J* = 8.4 Hz, 2H), 6.98-7.03 (m, 3H), 7.18 (d, *J* = 2.0 Hz, 1H), 7.29 (d, *J* = 9.3 Hz, 1H), 7.38 (d, *J* = 9.0 Hz, 1H), 7.63 (d, *J* = 9.0 Hz, 1H), 8.72 (s, 1H); ^{13}C NMR (100 MHz, $CDCl_3$), δ (ppm): 14.2, 20.3, 53.7, 62.6, 110.8, 112.0, 118.8, 119.8, 123.4, 123.8, 127.3, 127.7, 127.9, 130.0, 130.9, 144.2, 151.3, 151.9, 174.5; HRMS (ESI) m/z calculated for $C_{21}H_{21}NO_4$ $(M+H)^+$352.1543, Found 352.1544.

Ethyl 6-methyl-4-phenylquinoline-2-carboxylate (**3ah**) [5] White solid; mp: 172.2-172.3^{o}C;^{1}H NMR (400 MHz, $CDCl_3$), δ (ppm):1.50 (t, *J* = 7.2 Hz, 3H), 2.51 (s, 3H), 4.58 (q, *J* = 7.2 Hz, 2H), 7.53-7.57 (m, 5H), 7.63 (dd,*J*= 8.4, 1.6 Hz, 1H), 7.72 (s, 1H), 8.11(s, 1H), 8.29 (d, *J* = 8.8 Hz, 2H); ^{13}C NMR (100 MHz, $CDCl_3$), δ (ppm):14.4, 22.0, 62.7, 121.4, 124.4, 127.8, 128.6, 128.7, 129.5, 130.9, 132.3, 137.8, 138.9, 146.8, 146.9, 148.9, 165.6.

ethyl 6-methyl-4-(p-tolyl)quinoline-2-carboxylate (**3ai**) White solid; mp: 150-151°C; ^{1}H NMR (400 MHz, $CDCl_3$), δ (ppm):1.50 (t, *J* = 7.2 Hz, 3H), 2.50 (s, 3H), 2.51 (s, 3H), 4.58 (q, *J* = 7.2 Hz, 2H), 7.38 (d, *J*= 8.0 Hz, 5H), 7.44 (d, *J*= 8.0 Hz, 5H), 7.63 (dd, *J*= 8.4, 1.2 Hz, 1H), 7.75 (s,

1H), 8.10 (s, 1H), 8.28 (d, *J* = 8.4 Hz, 1H); ^{13}C NMR (100 MHz, $CDCl_3$), δ (ppm):14.4, 21.3, 22.0, 62.1, 121.4, 124.5, 127.9, 129.4, 129.5, 130.9, 132.2, 134.9, 138.6, 138.8, 146.8, 146.9, 149.0, 165.7.

ethyl 4-(4-fluorophenyl)-6-methylquinoline-2-carboxylate (**3aj**) White solid; mp: 174-175°C; ^{1}H NMR (400 MHz, $CDCl_3$), δ (ppm):1.51 (t, *J* = 7.2 Hz, 3H), 2.52 (s, 3H), 4.58 (q, *J* = 7.2 Hz, 2H), 7.38 (d, *J*= 8.0 Hz, 5H), 7.44 (d, *J*= 8.0 Hz, 5H), 7.63 (dd, *J*= 8.4, 1.2 Hz, 1H), 7.75 (s, 1H), 8.10 (s, 1H), 8.28 (d, *J* = 8.4 Hz, 1H); ^{13}C NMR (100 MHz, $CDCl_3$), δ (ppm):14.4, 22.0, 62.2, 115.8 (d, J_{CF} = 21.5 Hz), 121.4, 124.1, 127.8, 131.0, 131.2, 131.3, 132.4, 133.7(d, J_{CF} = 3.4 Hz), 139.2, 146.9(d, J_{CF} = 11.0 Hz),147.8, 163.0(d, J_{CF} = 247.0 Hz), 165.6.

Spectra of prepared compounds

^{1}H NMR spectra of ethyl 2-(4-tolylamino)-2-(2,4,6-trimethoxyphenyl)acetate (3aa) [2]

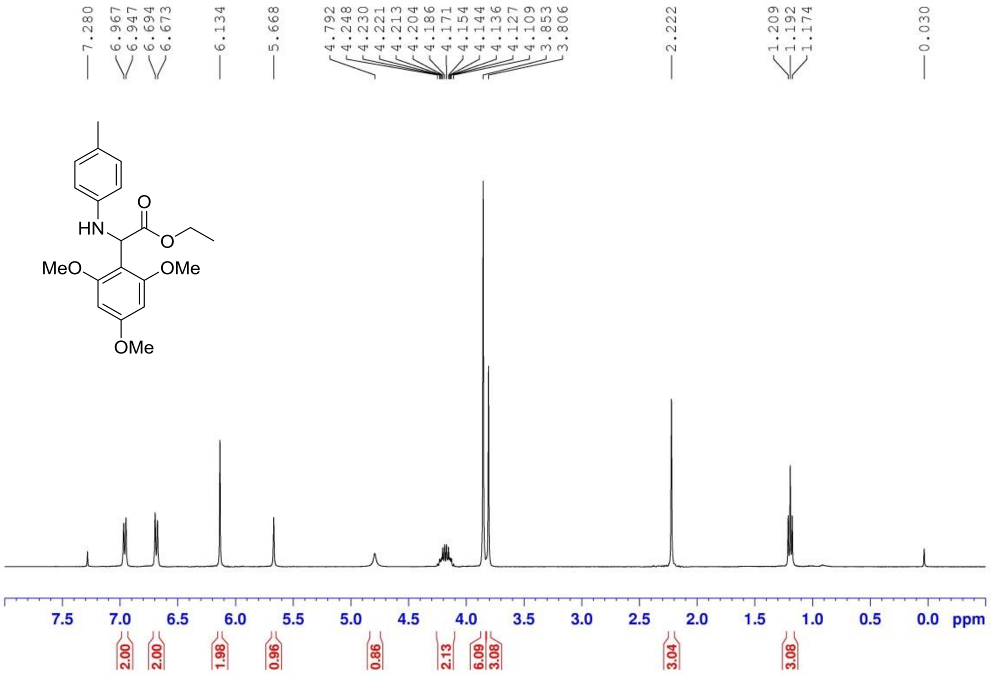

^{13}C NMR spectra of ethyl 2-(4-tolylamino)-2-(2,4,6-trimethoxyphenyl)acetate (3aa) [2]

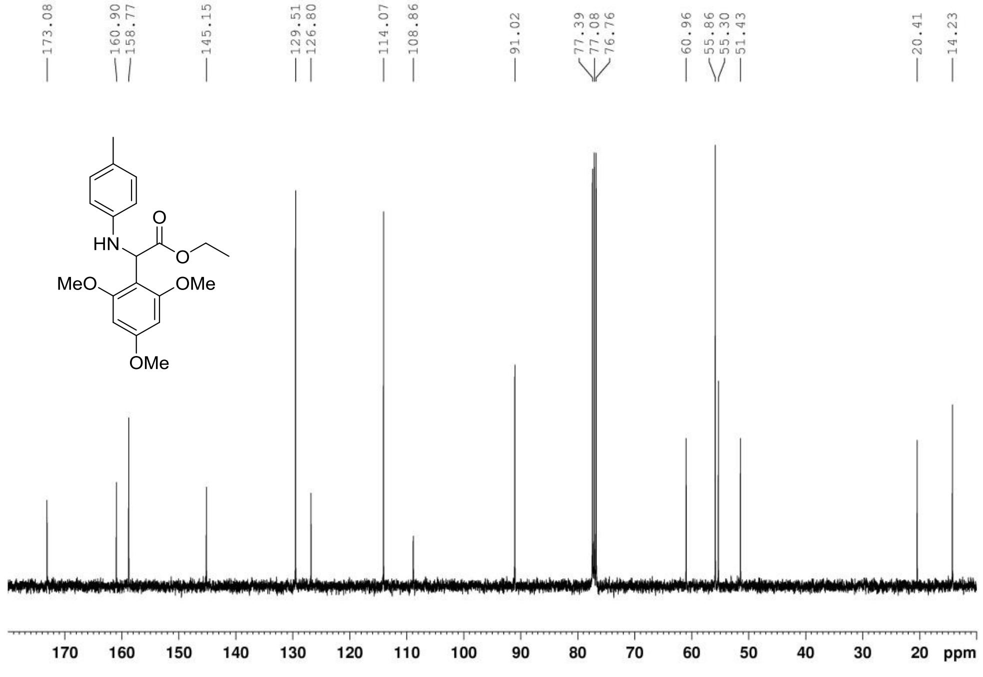

^{1}H NMR spectra of ethyl 2-((4-methoxyphenyl) amino)-2-(2,4,6-trimethoxyphenyl)acetate (3ba)

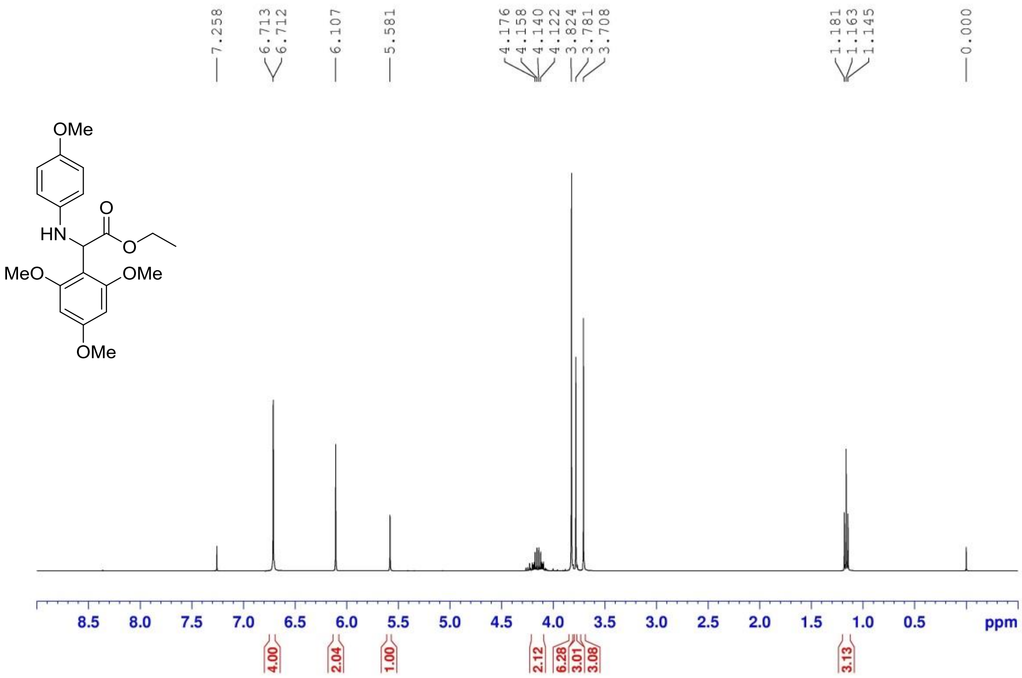

^{13}C NMR spectra of ethyl 2-((4-methoxyphenyl) amino)-2-(2,4,6-trimethoxyphenyl)acetate (3ba)

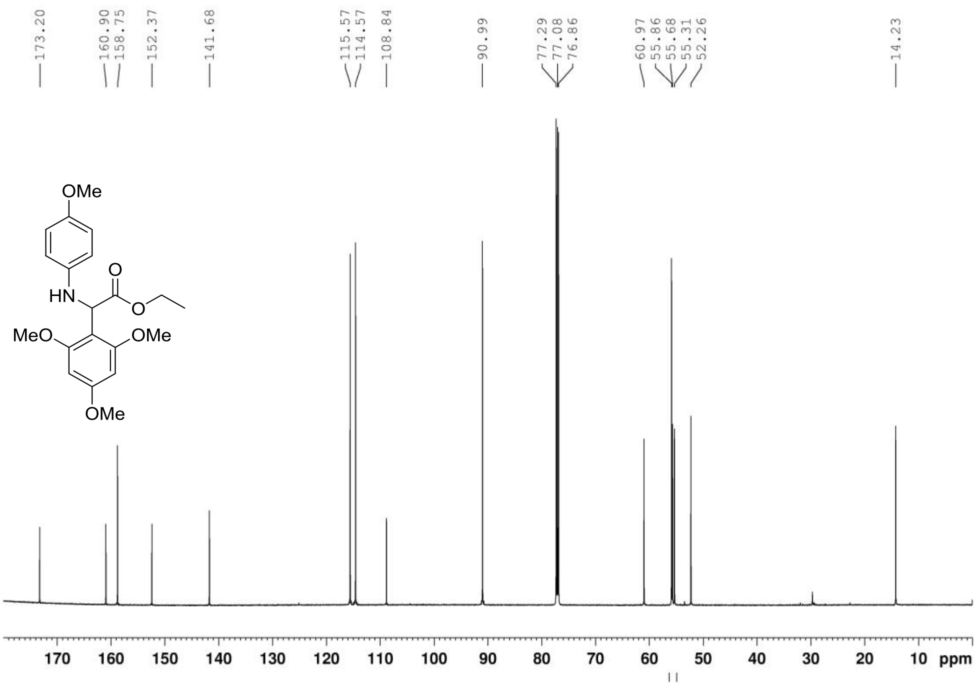

HRMS spectra of ethyl 2-((4-methoxyphenyl) amino)-2-(2,4,6-trimethoxyphenyl)acetate (3ba)

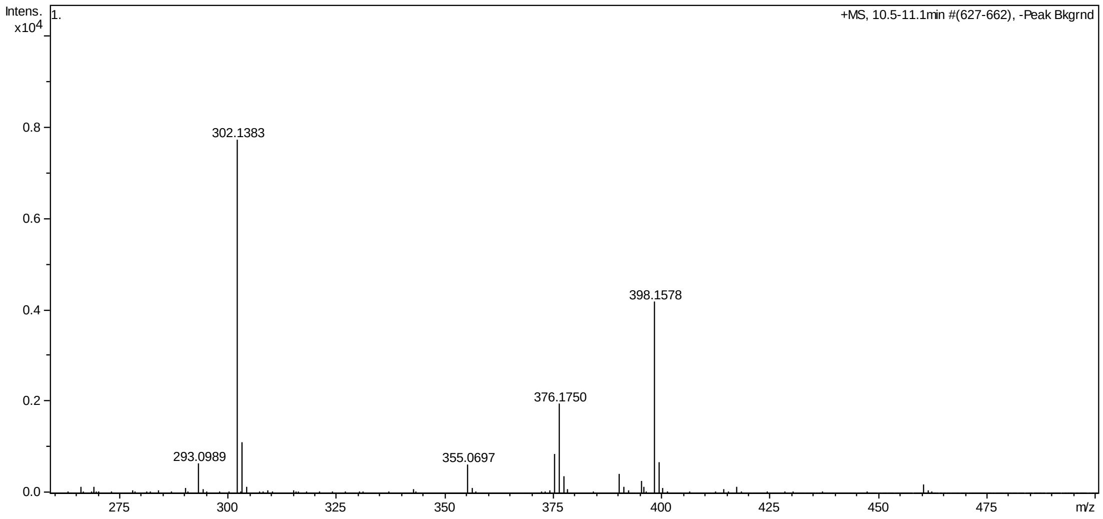

^{1}H NMR spectra of ethyl2-(4-bromophenylamino)-2-(2,4,6-trimethoxyphenyl)acetate (3ca) [2]

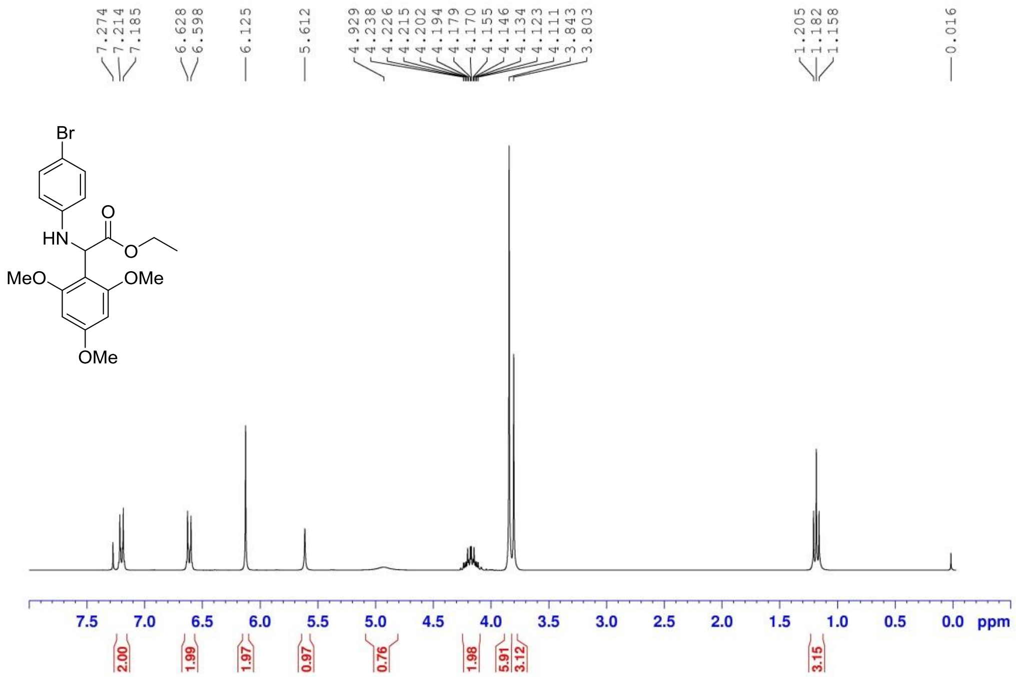

^{13}C NMR spectra of ethyl2-(4-bromophenylamino)-2-(2,4,6-trimethoxyphenyl)acetate (3ca) [2]

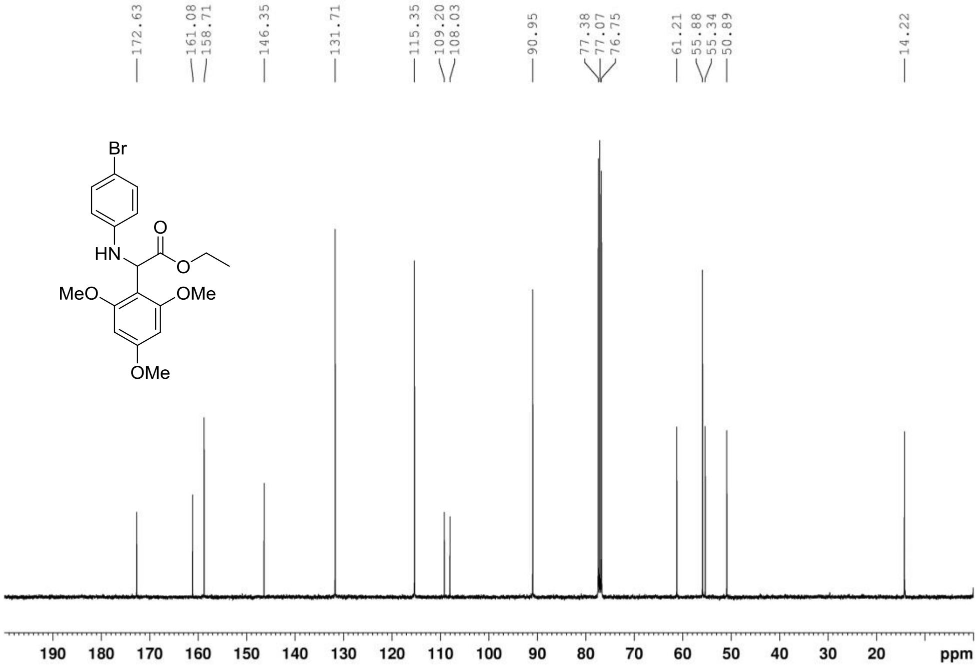

^{1}H NMR spectra of ethyl 2-(4-chlorophenylamino)-2-(2,4,6-trimethoxyphenyl)acetate (3da) [2]

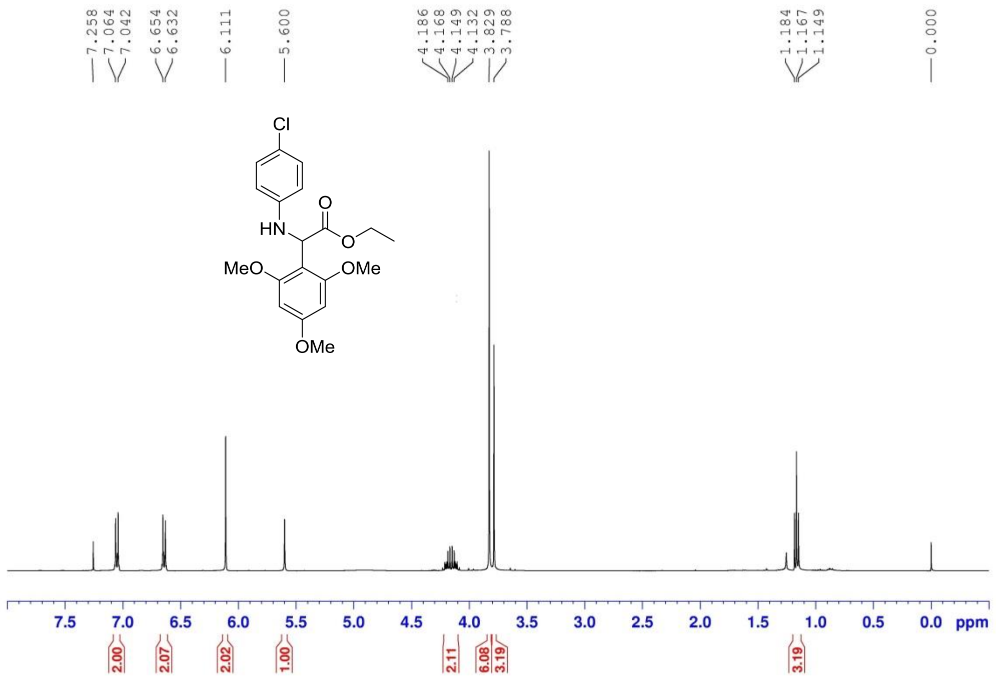

^{13}C NMR spectra of ethyl 2-(4-chlorophenylamino)-2-(2,4,6-trimethoxyphenyl)acetate (3da) [2]

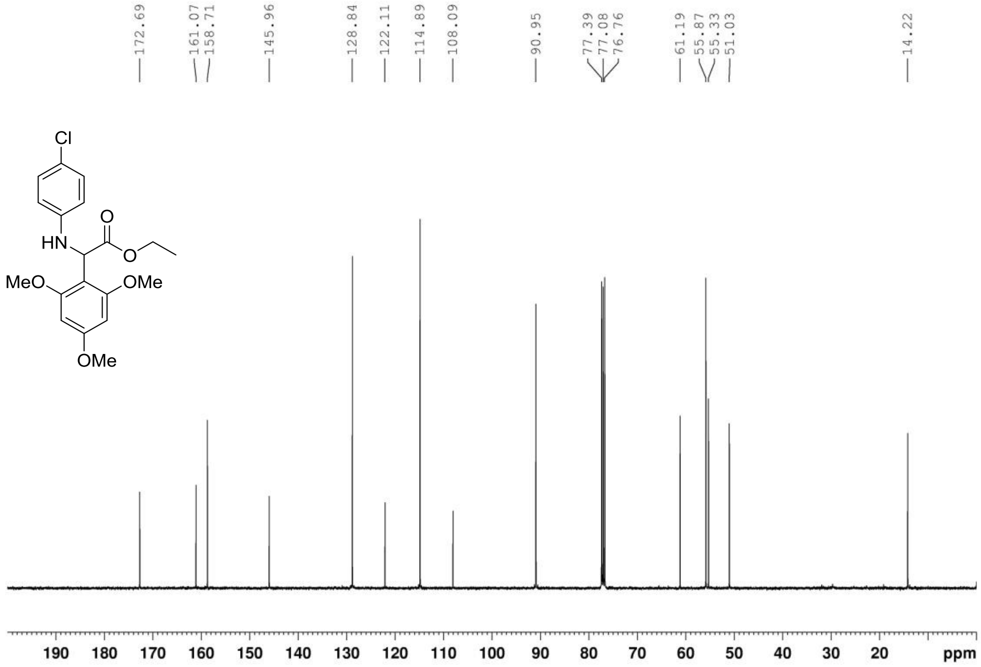

^{1}H NMR spectra of ethyl 2-((4-(trifluoromethyl)phenyl)amino)-2-(2,4,6-trimethoxyphenyl)acetate (3ea)

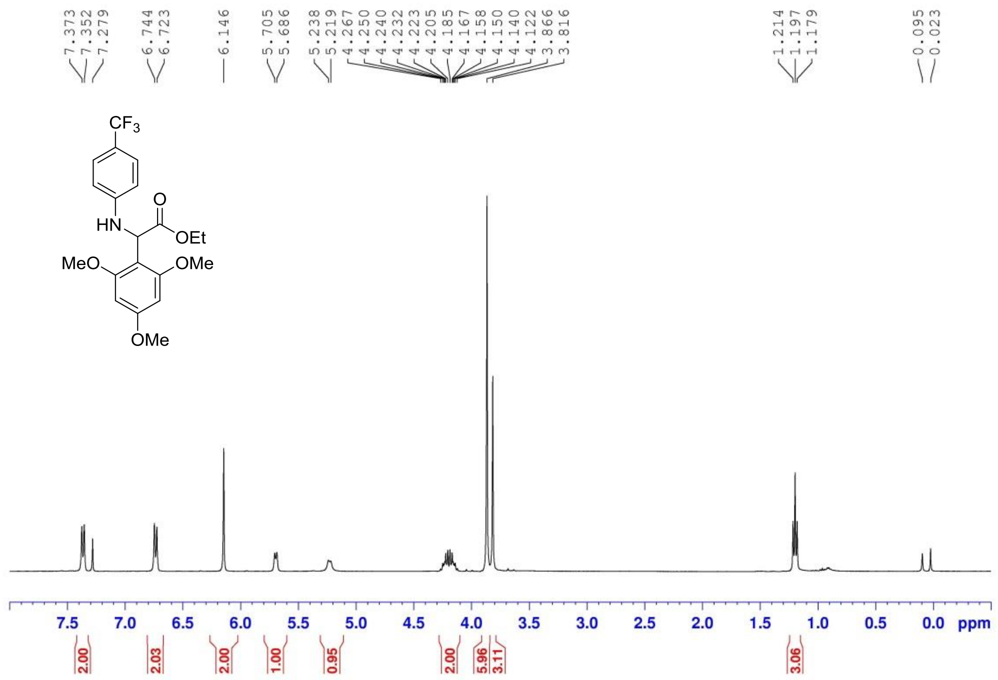

^{13}C NMR spectra of ethyl2-((4-(trifluoromethyl)phenyl)amino)-2-(2,4,6-trimethoxyphenyl)acetate (3ea)

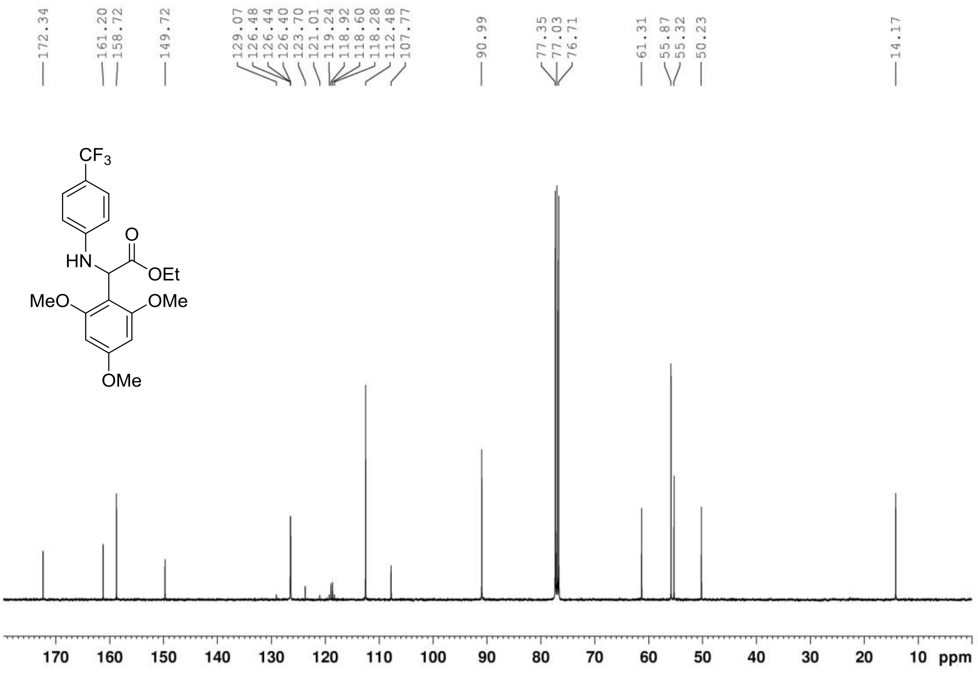

HRMS spectra of ethyl 2-((4-(trifluoromethyl)phenyl)amino)-2-(2,4,6-trimethoxyphenyl)acetate (3ea)

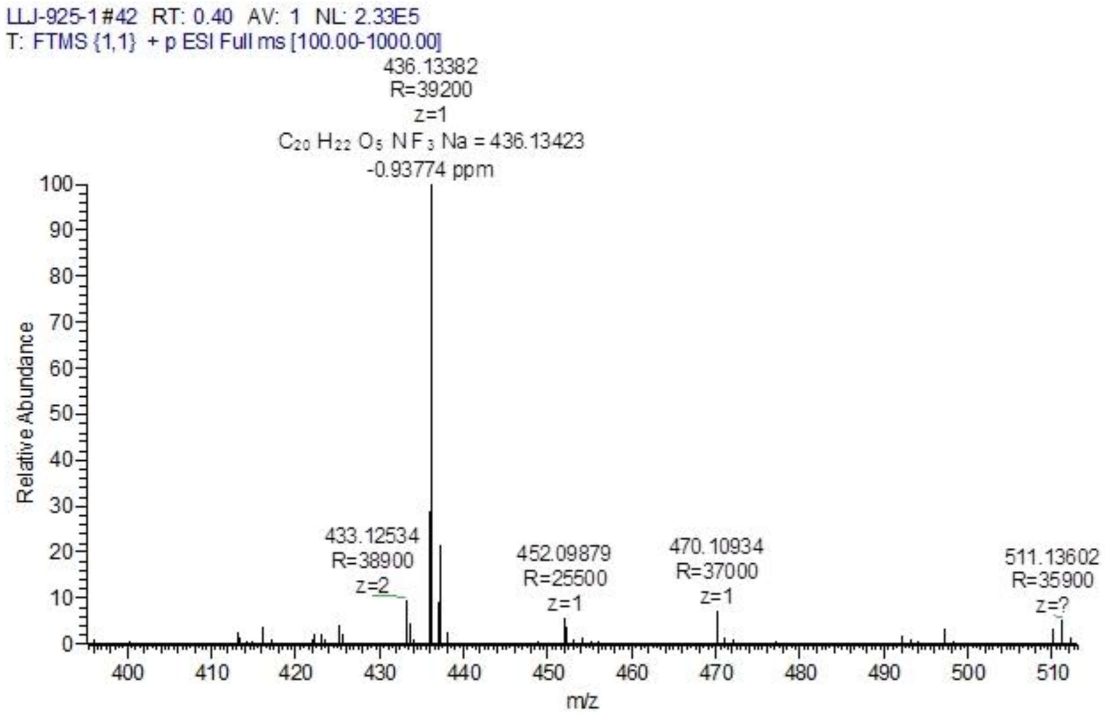

^{1}H NMR spectra of ethyl 2-(phenylamino)-2-(2,4,6-trimethoxyphenyl)acetate (3fa) [2]

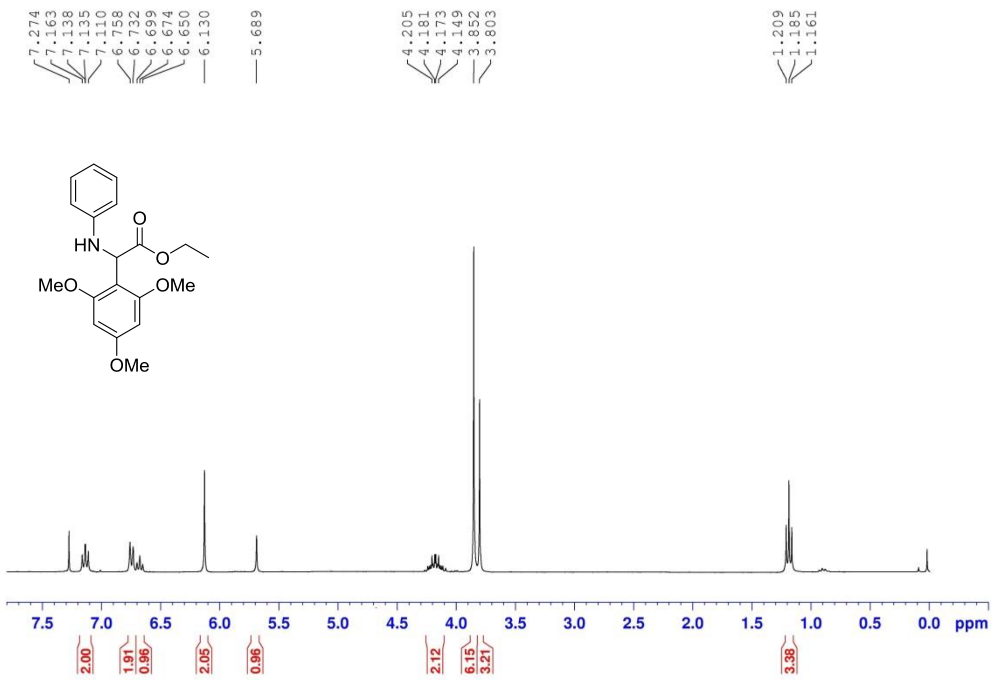

^{13}C NMR spectra of ethyl 2-(phenylamino)-2-(2,4,6-trimethoxyphenyl)acetate (3fa) [2]

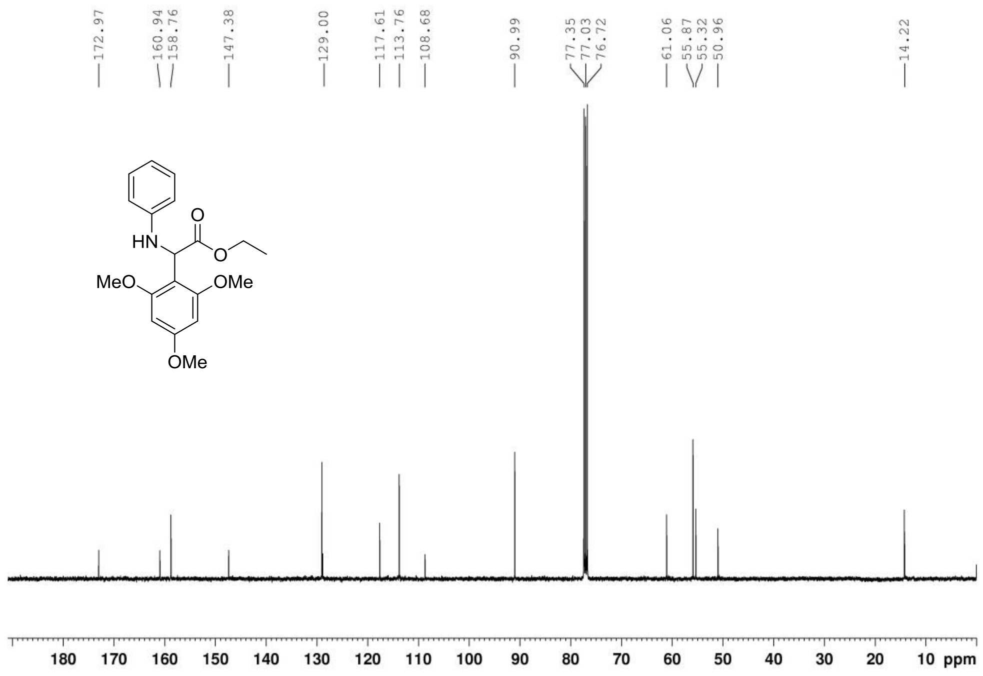

^{1}H NMR spectra of ethyl 2-(2-tolylamino)-2-(2,4,6-trimethoxyphenyl)acetate (3ga) [2]

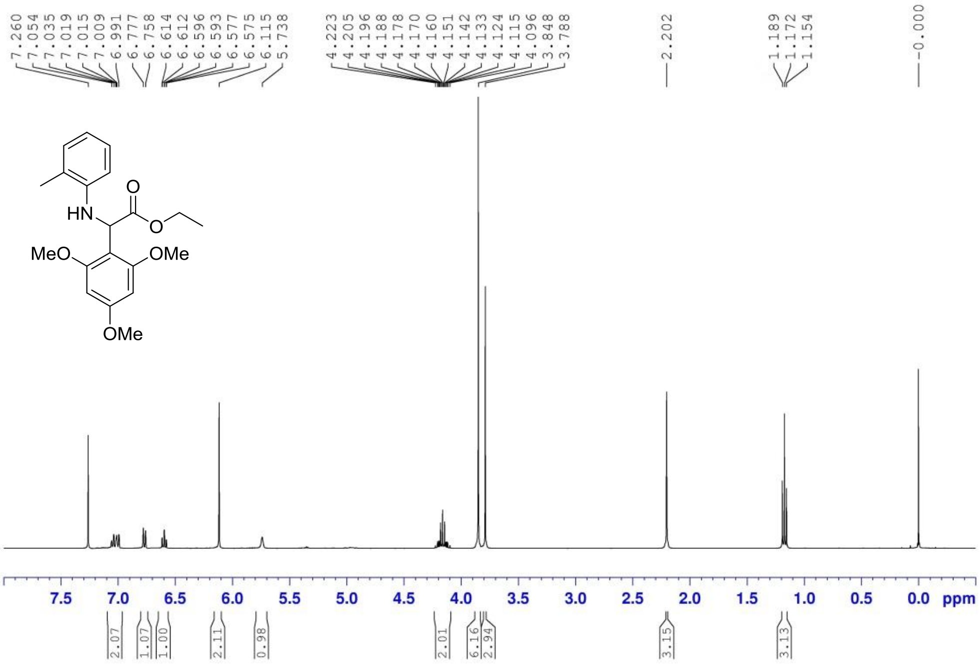

^{13}C NMR spectra of ethyl 2-(2-tolylamino)-2-(2,4,6-trimethoxyphenyl)acetate (3ga) [2]

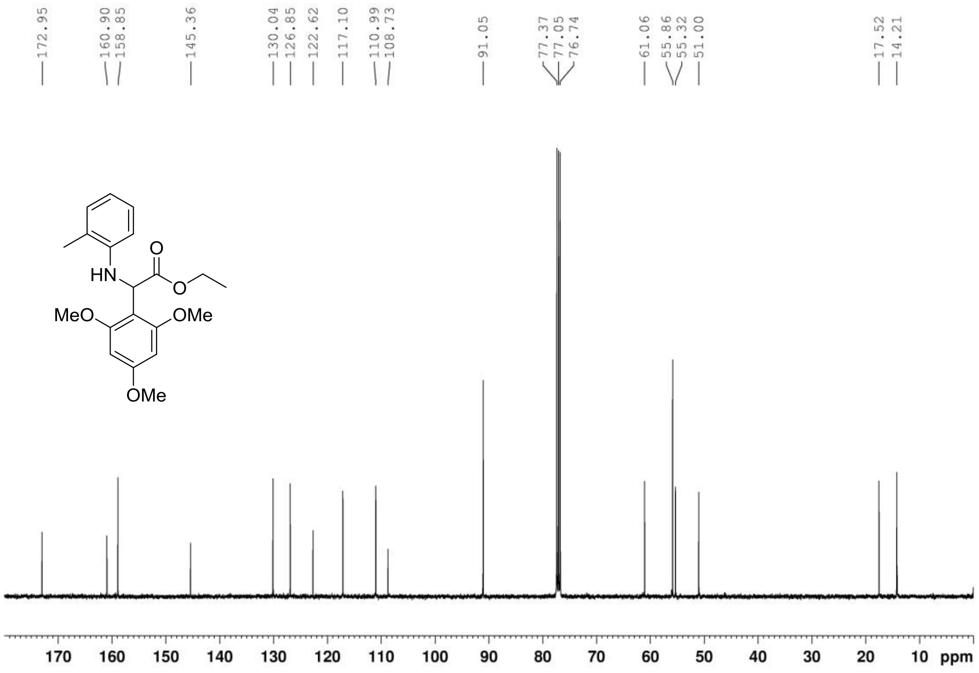

^{1}H NMR spectra of ethyl 2-(3-tolylamino)-2-(2,4,6-trimethoxyphenyl)acetate (3ha) [2]

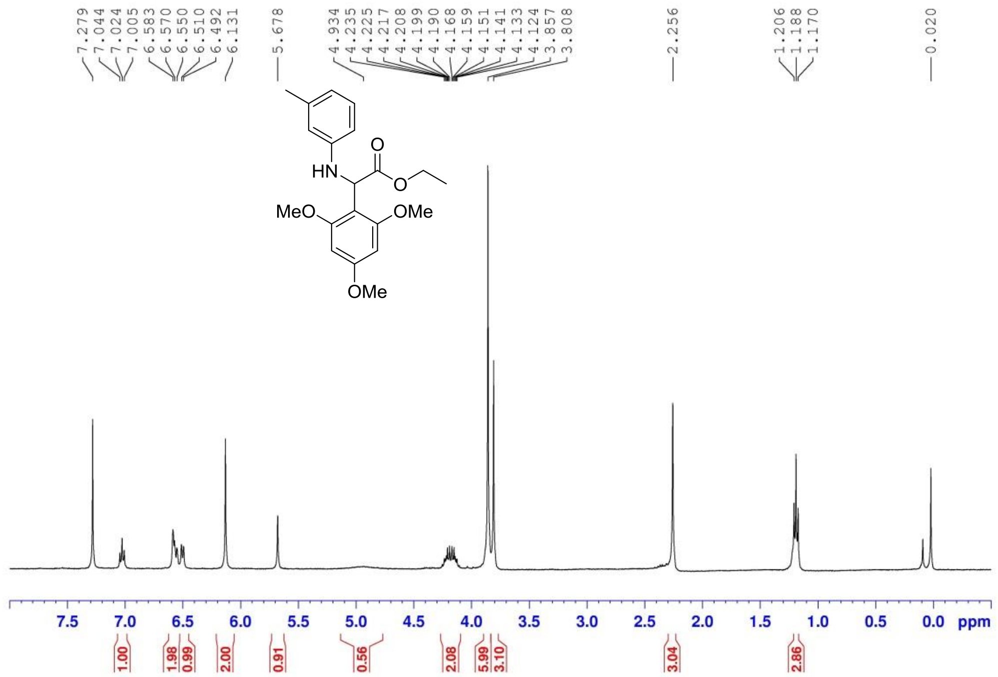

^{13}C NMR spectra of ethyl 2-(3-tolylamino)-2-(2,4,6-trimethoxyphenyl)acetate (3ha) [2]

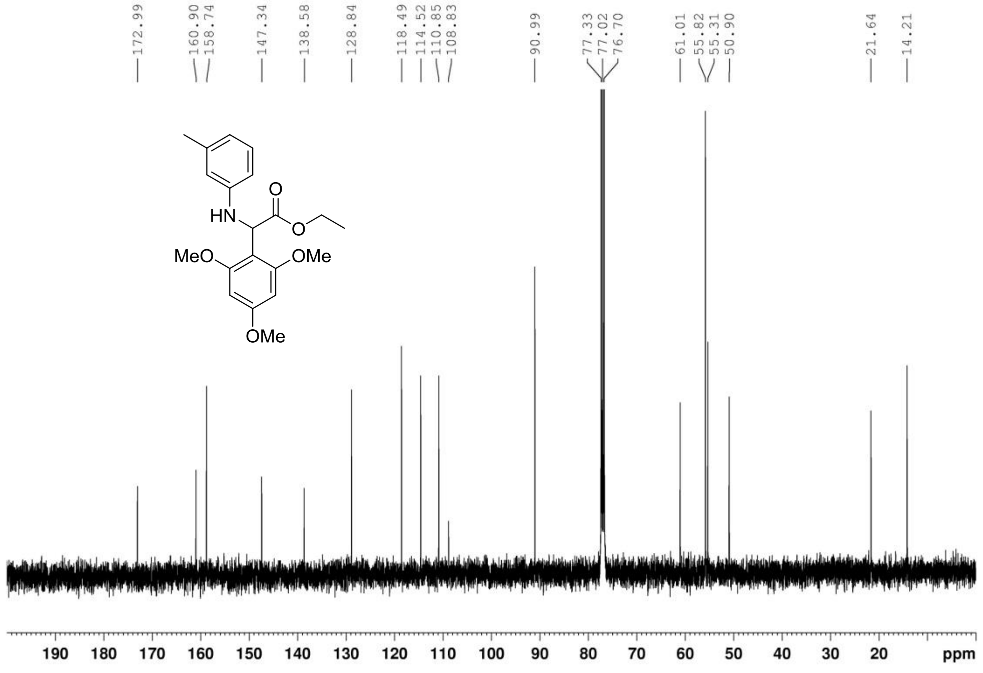

^{1}H NMR spectra of methyl 2-(4-tolylamino)-2-(2,4,6-trimethoxyphenyl)acetate (3ia) [2]

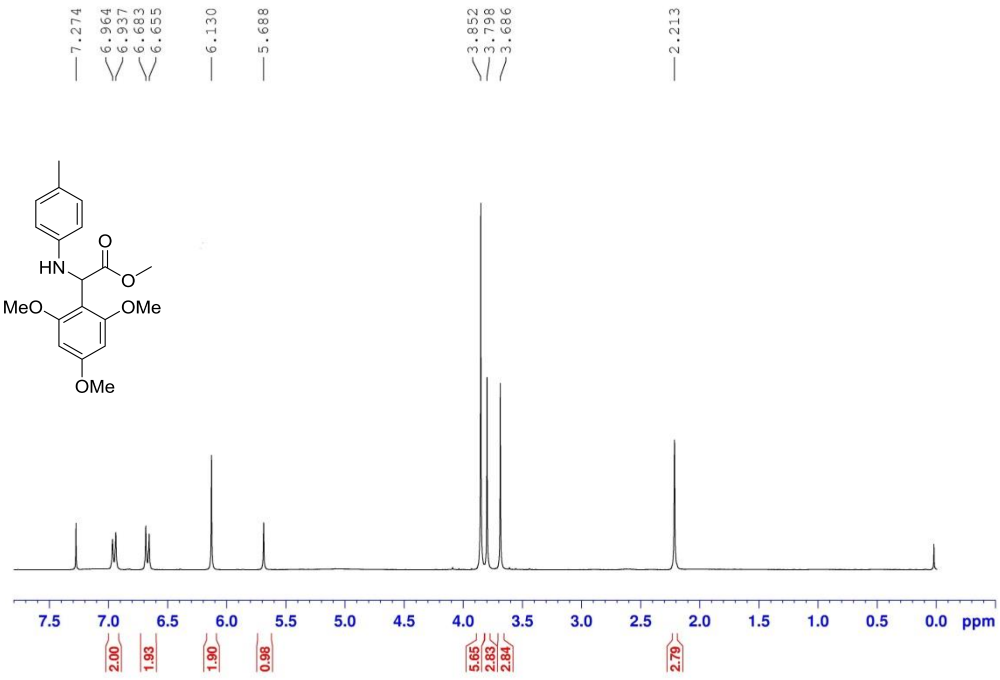

^{13}C NMR spectra of methyl 2-(4-tolylamino)-2-(2,4,6-trimethoxyphenyl)acetate (3ia) [2]

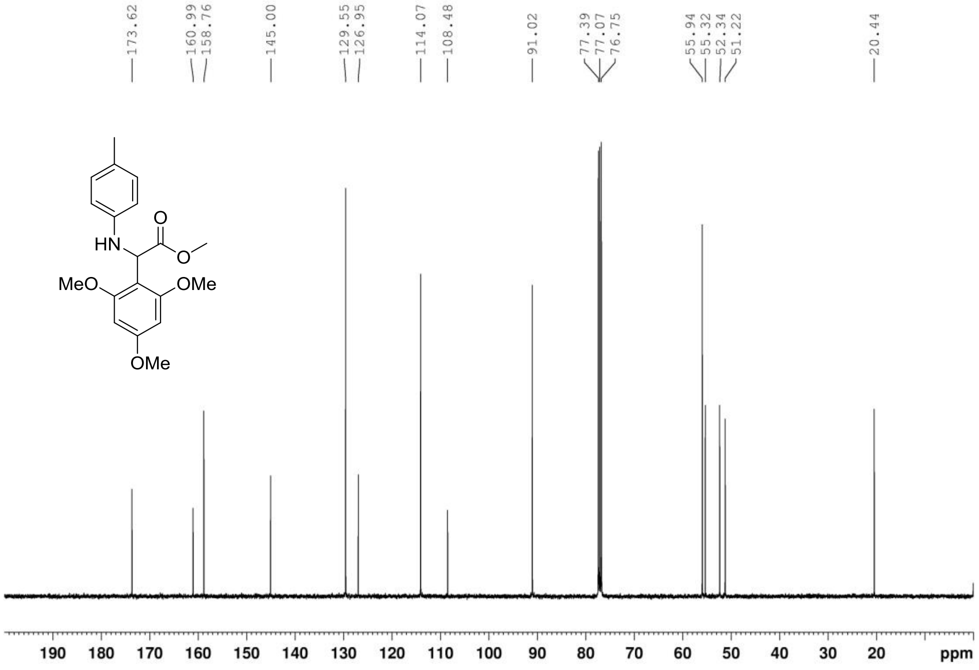

^{1}H NMR spectra of benzyl 2-(*p*-tolylamino)-2-(2,4,6-trimethoxyphenyl)acetate (3ja)

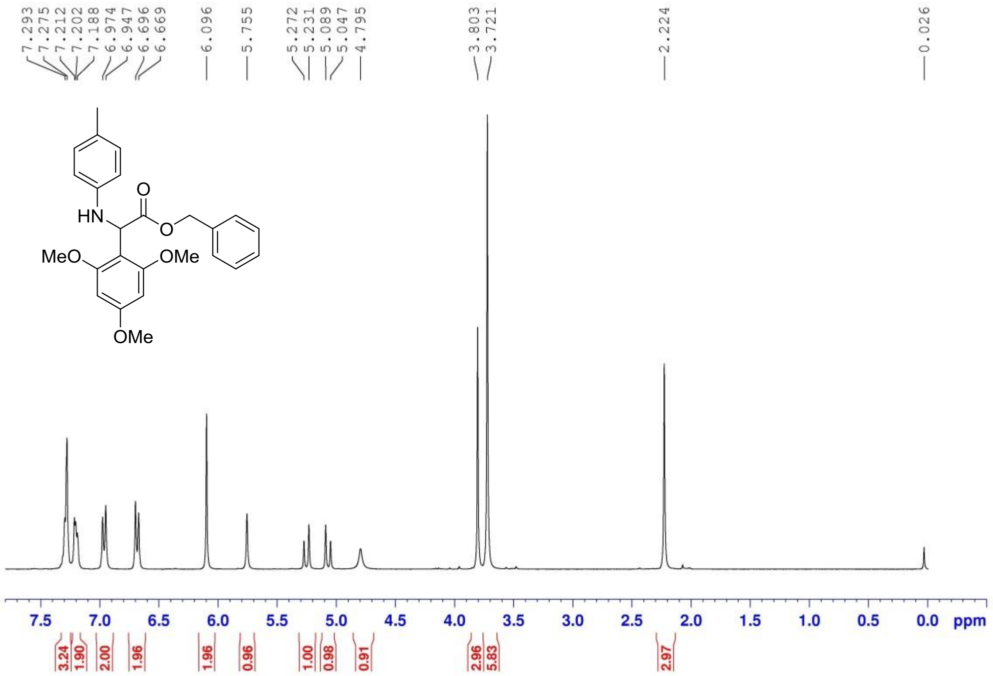

^{13}C NMR spectra of benzyl 2-(*p*-tolylamino)-2-(2,4,6-trimethoxyphenyl)acetate (3ja)

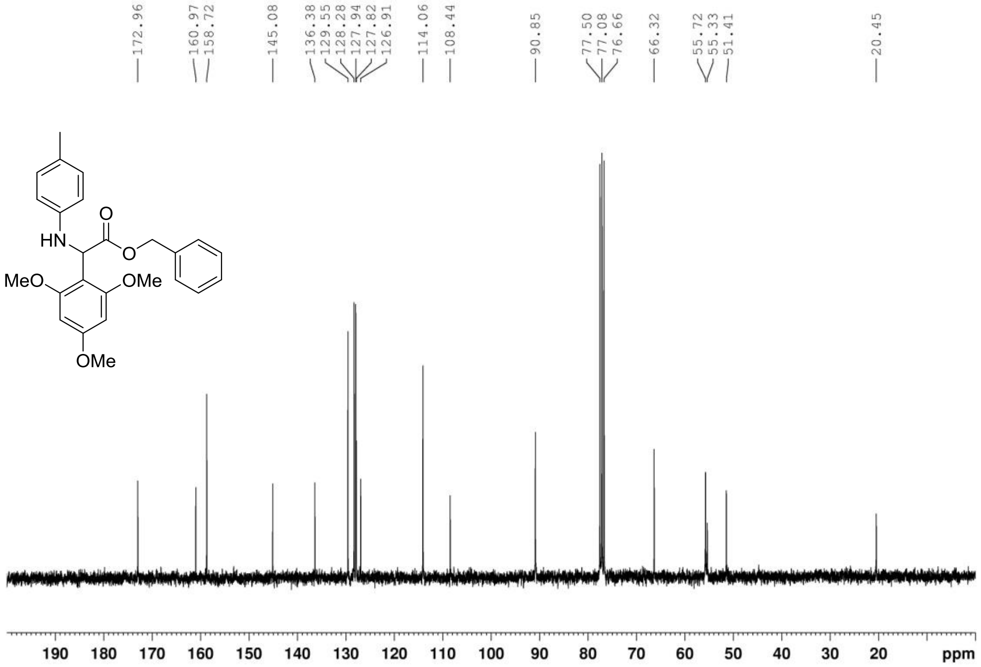

HRMS spectra of benzyl 2-(*p*-tolylamino)-2-(2,4,6-trimethoxyphenyl)acetate (3ja)

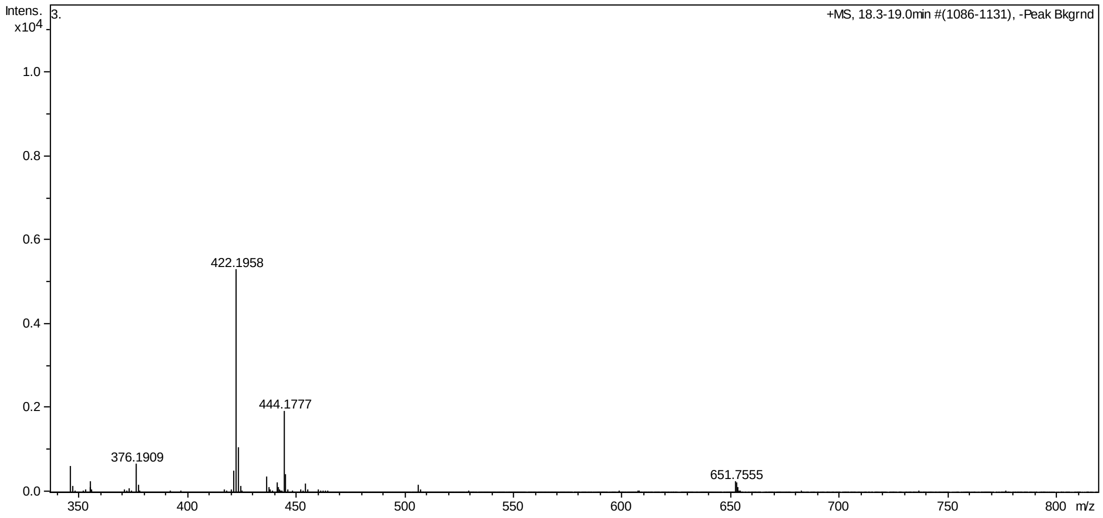

^{1}H NMR spectra of diethyl 2-benzoyl-3-(*p*-tolylamino)succinate (3ab) [3]

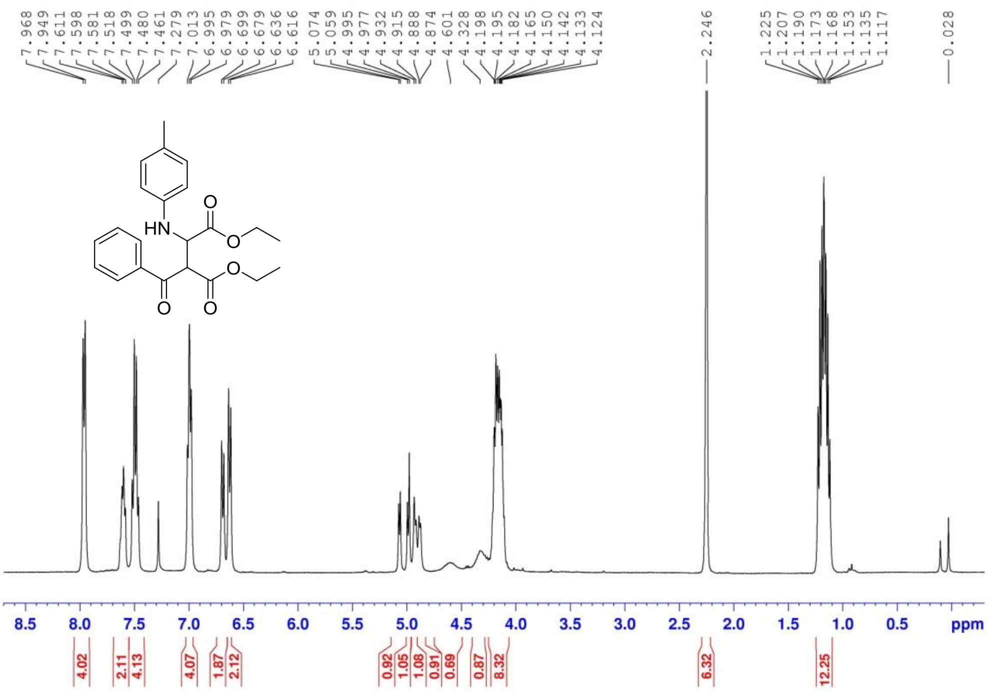

^{13}C NMR spectra of diethyl 2-benzoyl-3-(*p*-tolylamino)succinate (3ab) [3]

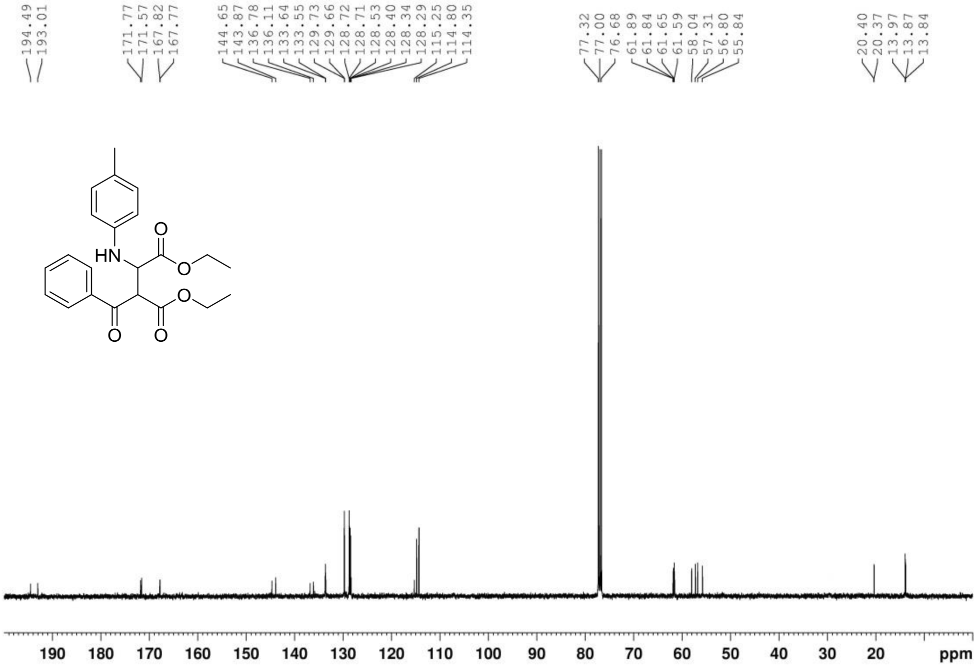

^{1}H NMR spectra of 2-ethyl 1,1-dimethyl 2-(*p*-tolylamino)ethane-1,1,2-tricarboxylate (3ac)

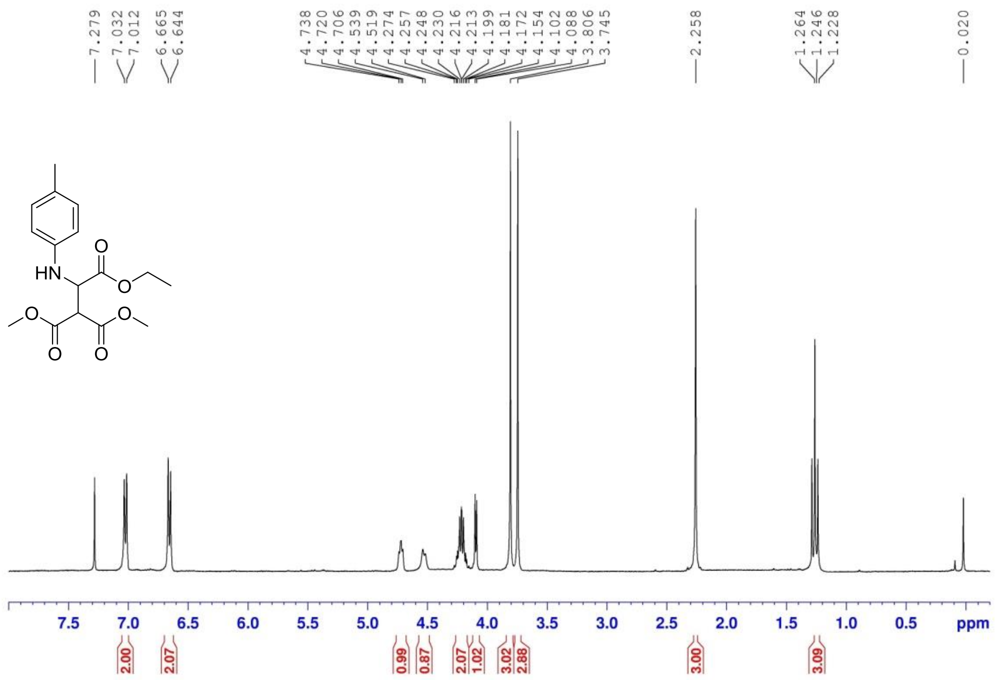

^{13}C NMR spectra of 2-ethyl 1,1-dimethyl 2-(*p*-tolylamino)ethane-1,1,2-tricarboxylate (3ac)

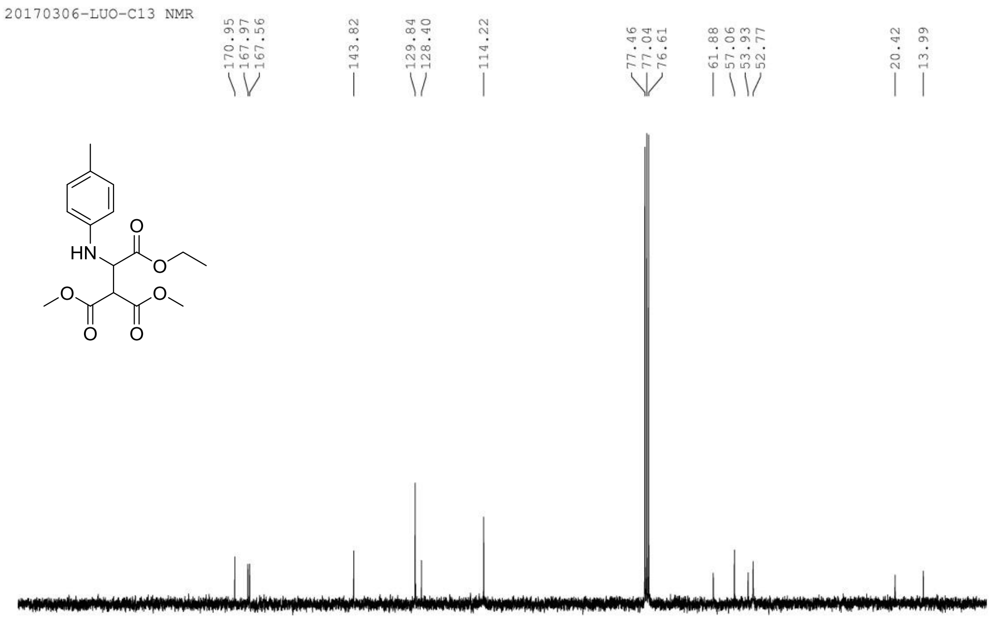

^{1}H NMR spectra of triethyl 2-(*p*-tolylamino)ethane-1,1,2-tricarboxylate (3ad) [4]

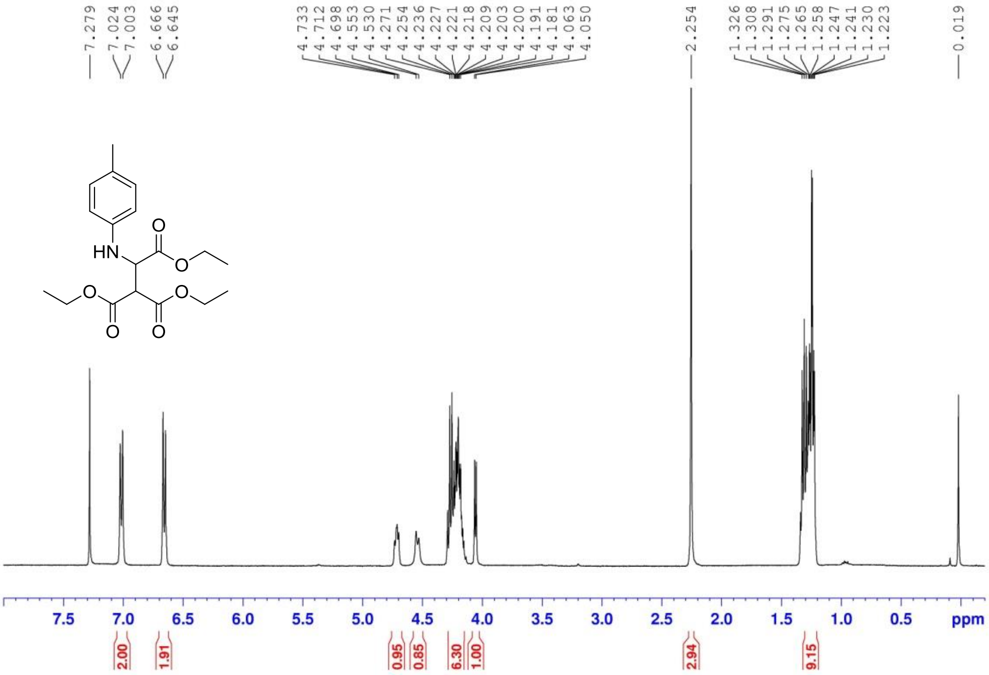

^{13}C NMR spectra of triethyl 2-(*p*-tolylamino)ethane-1,1,2-tricarboxylate (3ad) [4]

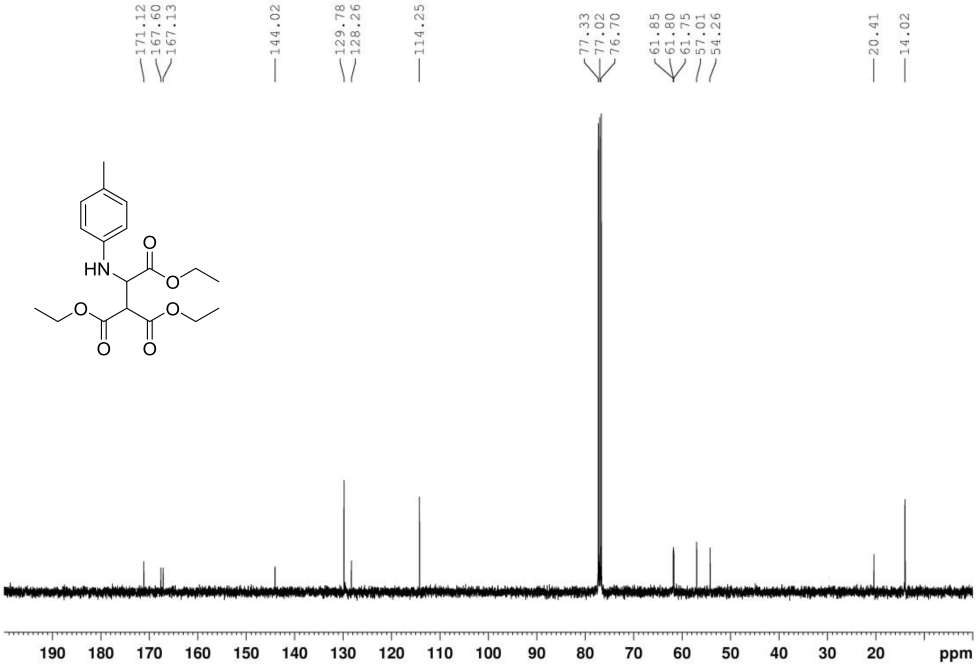

^{1}H NMR spectra of ethyl 2-(4-tolylamino)-2-(2-hydroxynaphthalen-1-yl)acetate (3ae) [2]

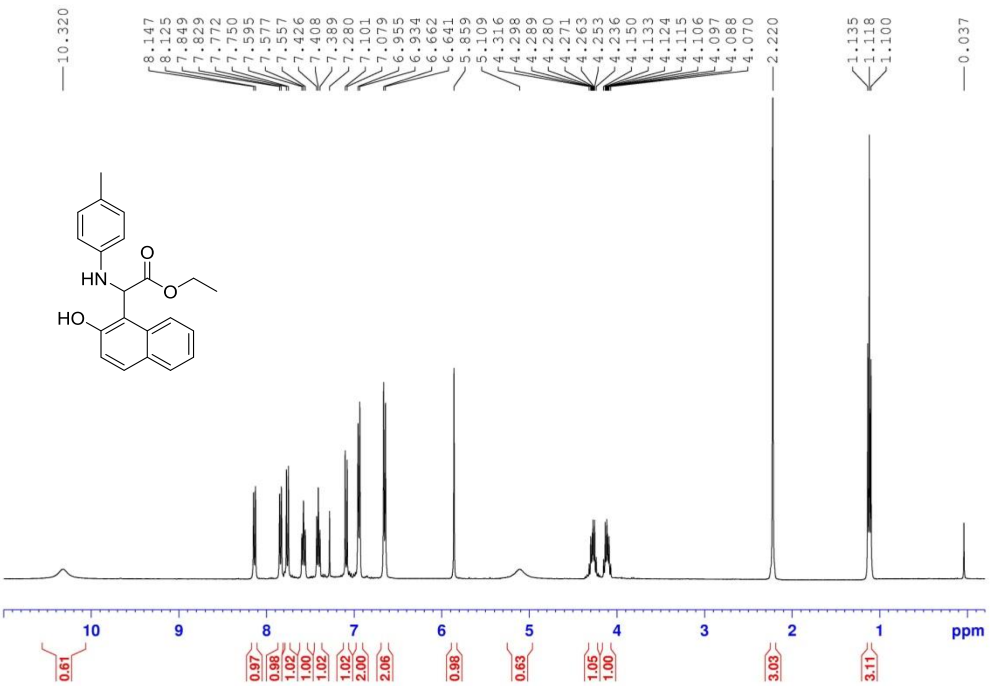

^{13}C NMR spectra of ethyl 2-(4-tolylamino)-2-(2-hydroxynaphthalen-1-yl)acetate (3ae) [2]

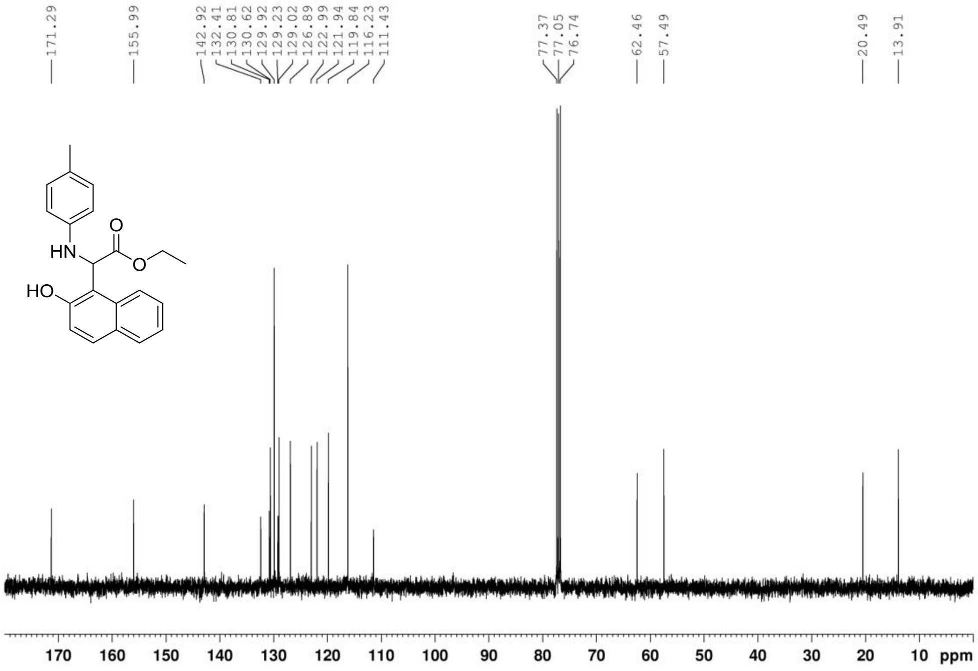

^{1}H NMR spectra of ethyl 2-(2,7-dihydroxynaphthalen-1-yl)-2-(*p*-tolylamino)acetate (3af)

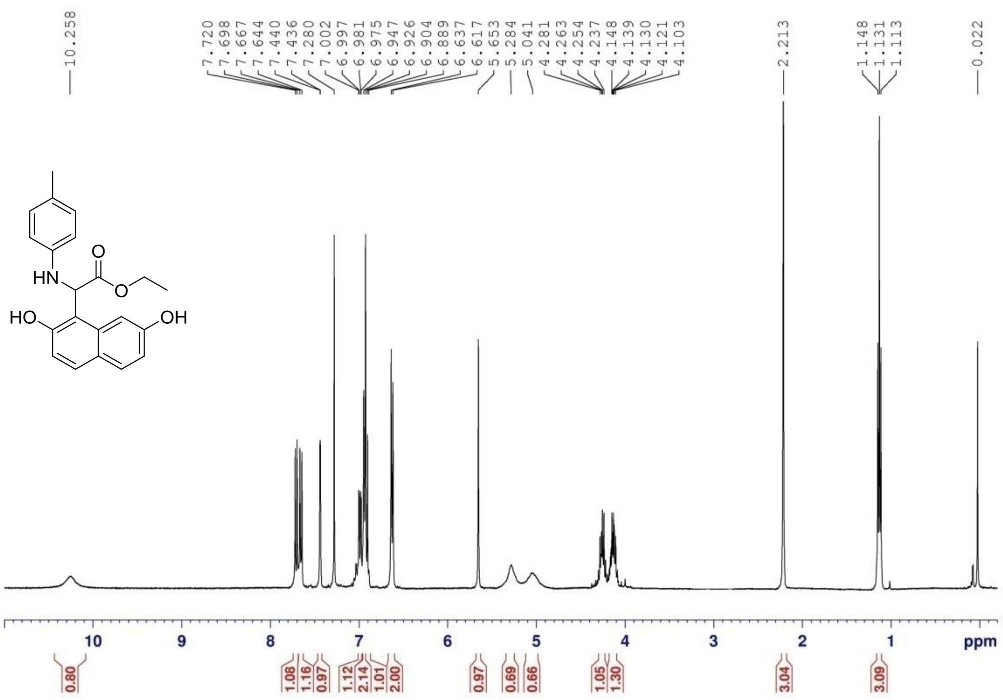

^{13}C NMR spectra of ethyl 2-(2,7-dihydroxynaphthalen-1-yl)-2-(*p*-tolylamino)acetate (3af)

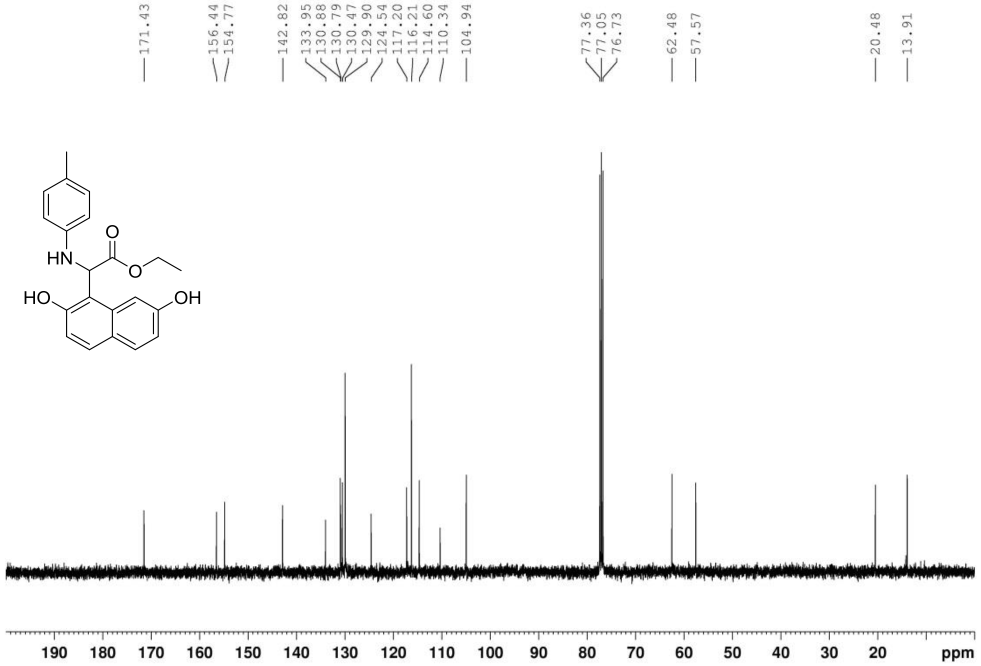

HRMS spectra of ethyl 2-(2,7-dihydroxynaphthalen-1-yl)-2-(*p*-tolylamino)acetate (3af)

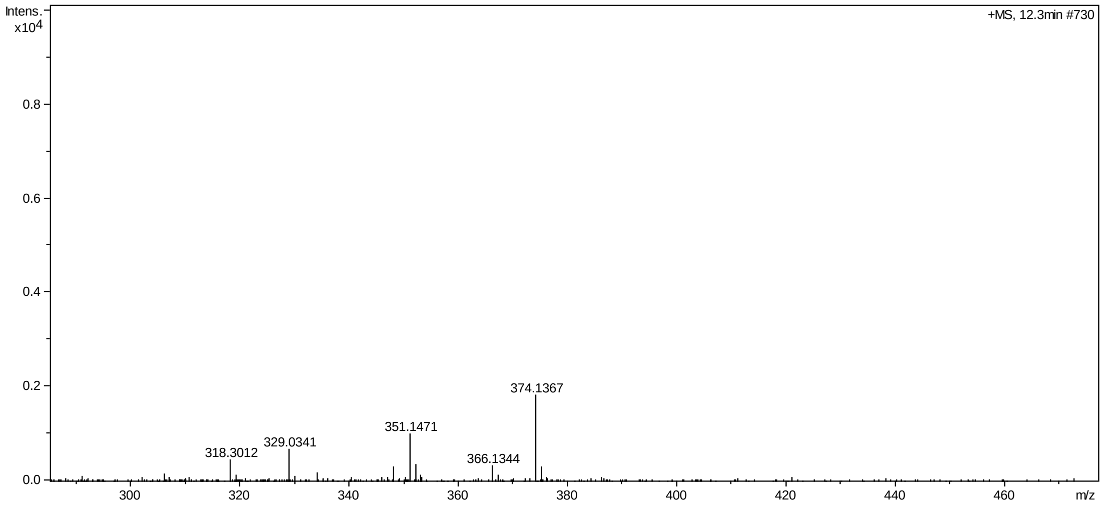

^{1}H NMR spectra of ethyl 2-(2,6-dihydroxynaphthalen-1-yl)-2-(*p*-tolylamino)acetate (3ag)

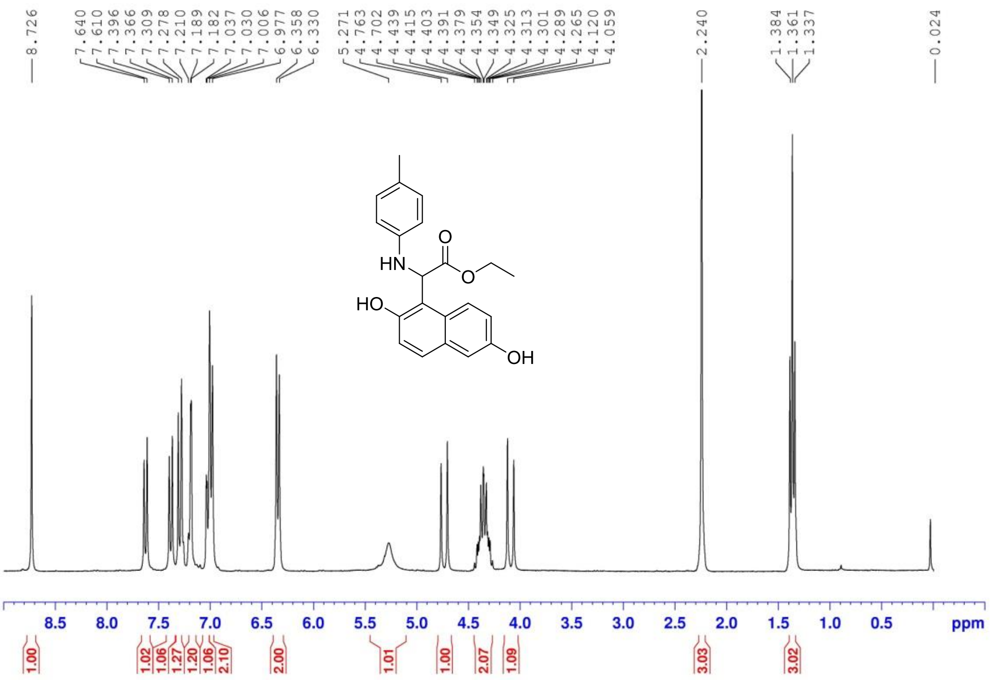

^{13}C NMR spectra of ethyl 2-(2,6-dihydroxynaphthalen-1-yl)-2-(*p*-tolylamino)acetate (3ag)

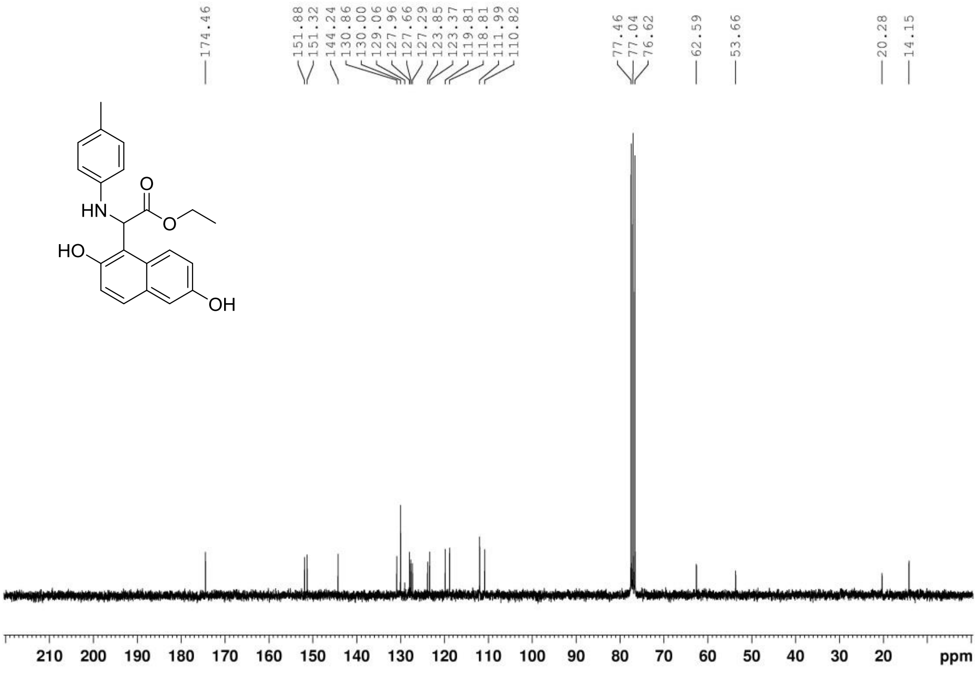

HRMS spectra of ethyl 2-(2,6-dihydroxynaphthalen-1-yl)-2-(*p*-tolylamino)acetate (3ag)

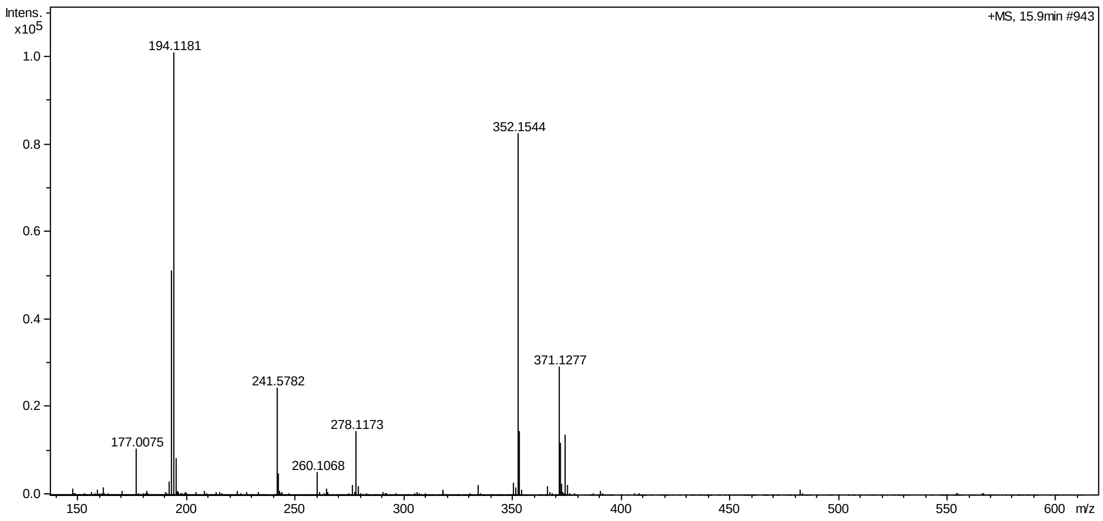

^{1}H NMR spectra of ethyl 6-methyl-4-phenylquinoline-2-carboxylate (3ah) [5]

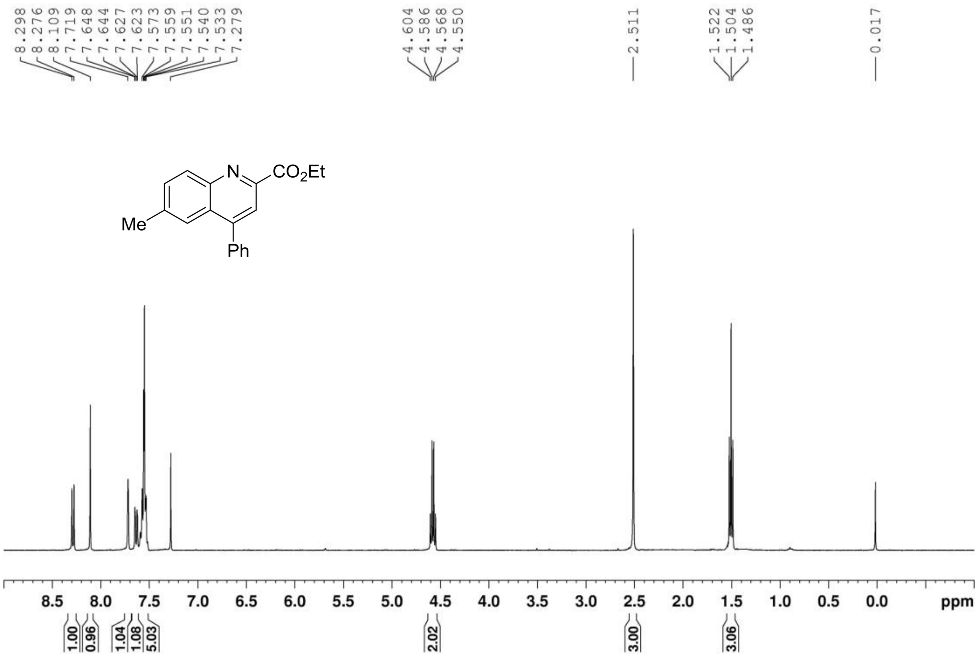

^{13}C NMR spectra of ethyl 6-methyl-4-phenylquinoline-2-carboxylate (3ah) [5]

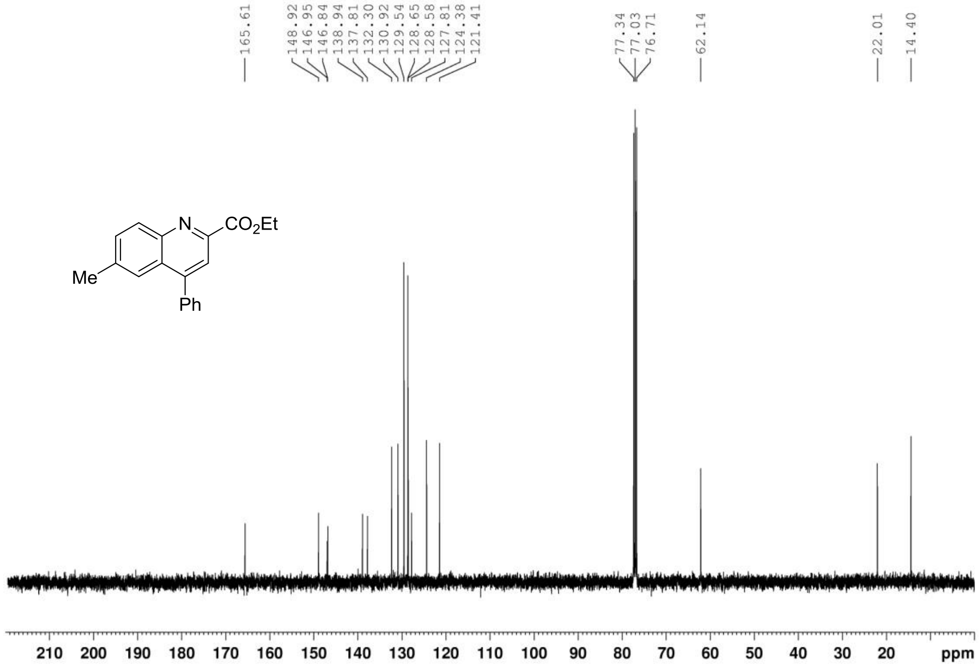

^{1}H NMR spectra of ethyl 6-methyl-4-(*p*-tolyl)quinoline-2-carboxylate (3ai) [6]

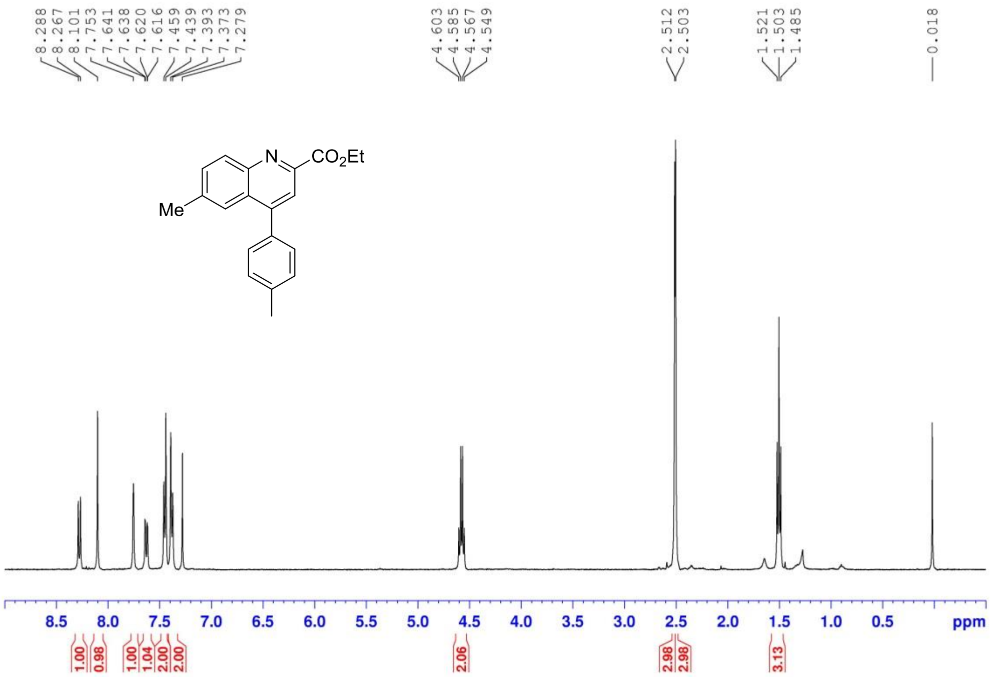

^{13}C NMR spectra of ethyl 6-methyl-4-(p-tolyl)quinoline-2-carboxylate (3ai) [6]

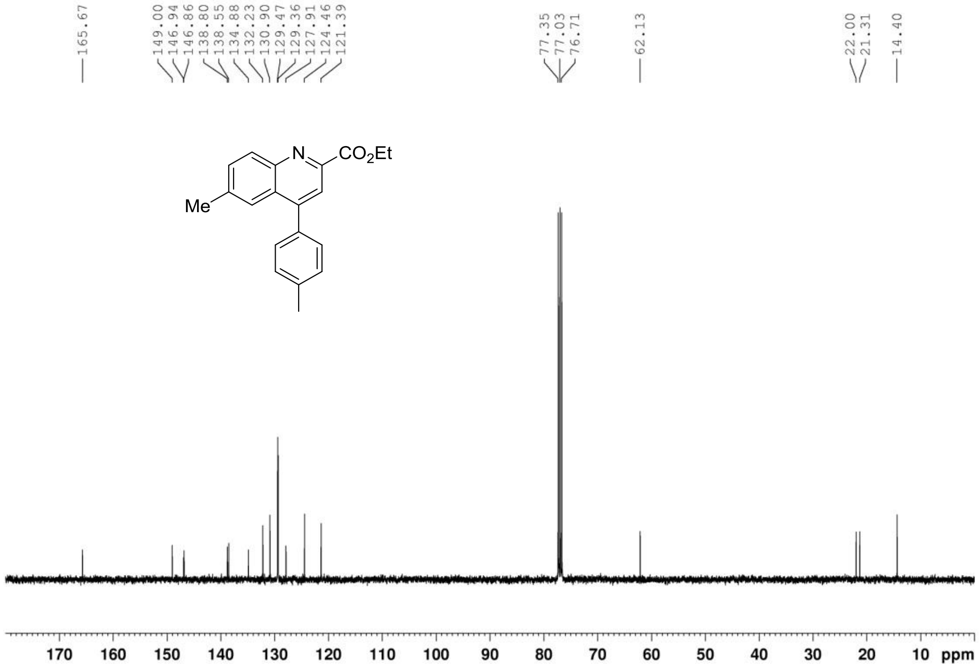

^{1}H NMR spectra of ethyl 4-(4-fluorophenyl)-6-methylquinoline-2-carboxylate (3aj) [6]

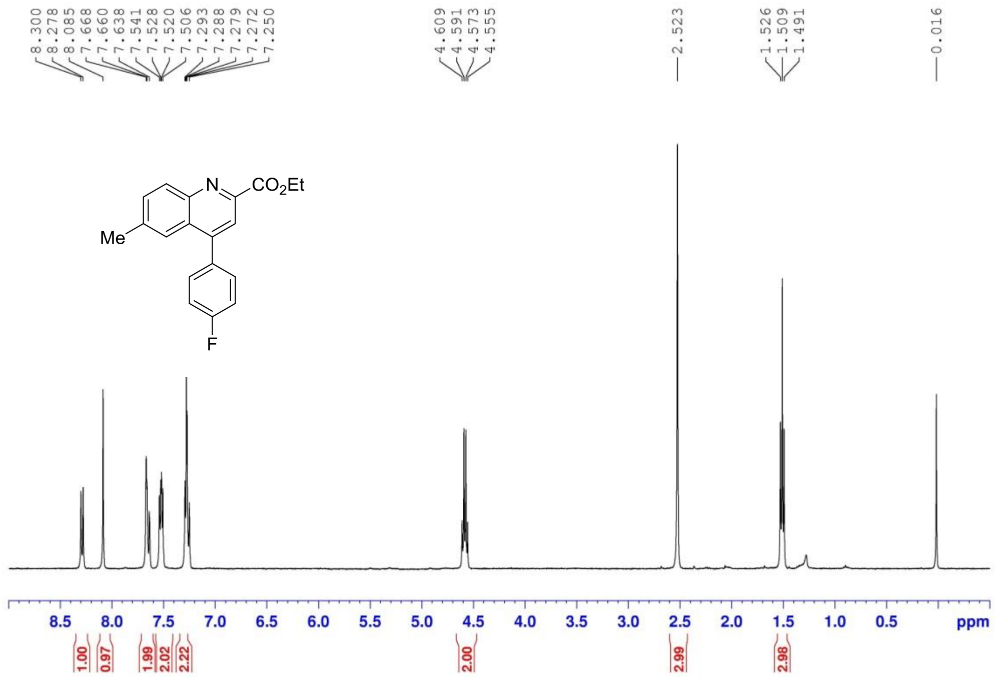

^{13}C NMR spectra of ethyl 4-(4-fluorophenyl)-6-methylquinoline-2-carboxylate (3aj) [6]

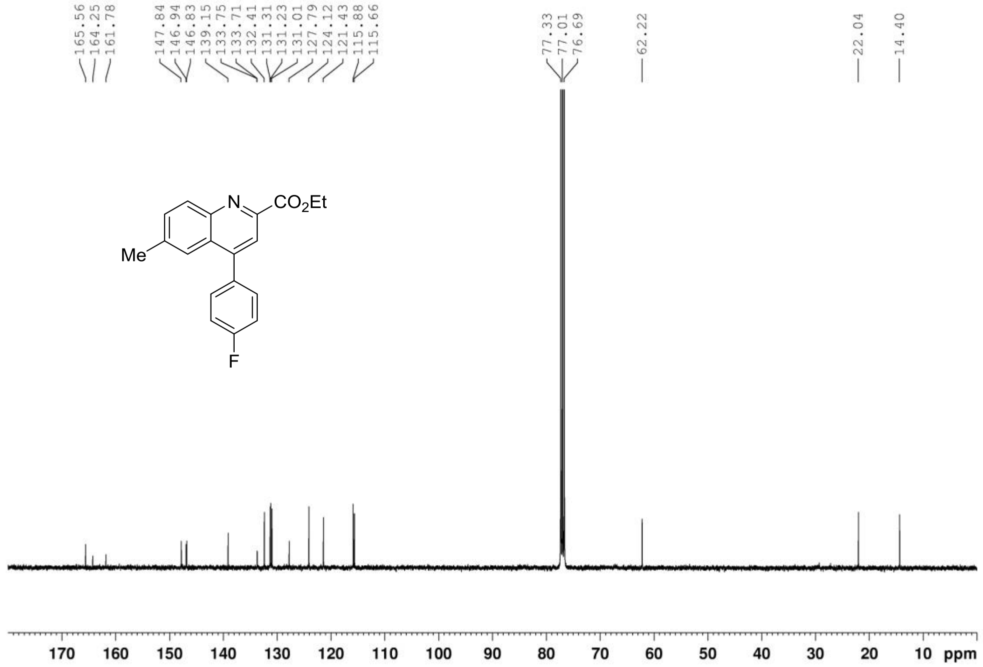

References

1 Rohlmann, R.; Stopka, T.; Richter, H.; Mancheno, O. G. *J. Org. Chem.***2013**, *78*, 6050.

2 Salman, M.; Zhu, Z.-Q.; Huang, Z.-Z. *Org. Lett.***2016**, *18*, 1526.

3 Wu, L.-Z.; Gao, X.-W.; Meng, Q.-Y.; Lei, T.; Zhong, J.-J.; Xiang, M.; Tong, Z.-H. Synthesis method using visible-light catalysis for secondary amine α-alkylation CN 104230734-A.

4 Chen, X.-M.; Li, X.-S.; Chan, A. S. C. *Chin. Chem. Lett.***2009**, *20*, 407.

5 Ni, M.-J.; Zhang, Y.; Gong, T.-T.; Feng, B.-N. *Adv. Synth. Catal.***2017**, *359*, 824.

6 Jia, X.; Peng, F.; Qing, C.; Huo, C.; Wang, X. *Org. Lett.* **2012**, *14*, 4030
